# Interacting hepatic PAI-1/tPA gene regulatory pathways influence impaired fibrinolysis severity in obesity

**DOI:** 10.1172/JCI135919

**Published:** 2020-07-13

**Authors:** Ze Zheng, Keiko Nakamura, Shana Gershbaum, Xiaobo Wang, Sherry Thomas, Marc Bessler, Beth Schrope, Abraham Krikhely, Rui-Ming Liu, Lale Ozcan, José A. López, Ira Tabas

**Affiliations:** 1 Department of Medicine, Columbia University Irving Medical Center, New York, New York, USA.; 2 Graduate School of Medicine and; 3 Faculty of Medicine, University of Tokyo, Tokyo, Japan.; 4 Neuroscience and Behavior Department, Barnard College, New York, New York, USA.; 5 Department of Surgery, Columbia University Irving Medical Center, New York, New York, USA.; 6 Division of Pulmonary Allergy, and Critical Care Medicine, Department of Medicine, University of Alabama at Birmingham, Birmingham, Alabama, USA.; 7 Department of Medicine, University of Washington, Seattle, Washington, USA.; 8 Bloodworks Research Institute, Seattle, Washington, USA.; 9 Department of Physiology and; 10 Department of Pathology and Cell Biology, Columbia University Irving Medical Center, New York, New York, USA.

**Keywords:** Hematology, Metabolism, Coagulation, Obesity

## Abstract

Fibrinolysis is initiated by tissue-type plasminogen activator (tPA) and inhibited by plasminogen activator inhibitor 1 (PAI-1). In obese humans, plasma PAI-1 and tPA proteins are increased, but PAI-1 dominates, leading to reduced fibrinolysis and thrombosis. To understand tPA–PAI-1 regulation in obesity, we focused on hepatocytes, a functionally important source of tPA and PAI-1 that sense obesity-induced metabolic stress. We showed that obese mice, like humans, had reduced fibrinolysis and increased plasma PAI-1 and tPA, due largely to their increased hepatocyte expression. A decrease in the PAI-1 (*SERPINE1*) gene corepressor Rev-Erbα increased PAI-1, which then increased the tPA gene *PLAT* via a PAI-1/LRP1/PKA/p-CREB1 pathway. This pathway was partially counterbalanced by increased DACH1, a *PLAT*-negative regulator. We focused on the PAI-1/*PLAT* pathway, which mitigates the reduction in fibrinolysis in obesity. Thus, silencing hepatocyte PAI-1, CREB1, or tPA in obese mice lowered plasma tPA and further impaired fibrinolysis. The PAI-1/*PLAT* pathway was present in primary human hepatocytes, and associations among PAI-1, tPA, and *PLAT* in livers from obese and lean humans were consistent with these findings. Knowledge of PAI-1 and tPA regulation in hepatocytes in obesity may suggest therapeutic strategies for improving fibrinolysis and lowering the risk of thrombosis in this setting.

## Graphical abstract

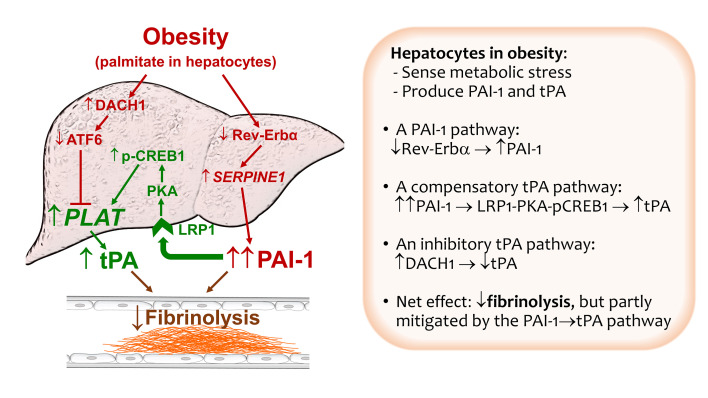

## Introduction

Obesity, which has now reached epidemic proportions, increases risks for arterial, venous, and microvascular thrombosis, including coronary thrombosis (1, 2), stroke (3), deep vein thrombosis (4, 5), pulmonary embolism (5, 6), and thrombotic thrombocytopenic purpura (5, 7). A major contributor to increased thrombosis in obesity is a reduction of fibrinolysis (8, 9), the primary mechanism that dissolves a blood clot (10). Tissue-type plasminogen activator (tPA) initiates fibrinolysis by converting the zymogen plasminogen to plasmin, a serine protease that degrades the fibrin clot (11, 12). We have shown in lean mice that hepatocytes maintain a basal level of circulating tPA that influences fibrinolysis following vessel injury (13). As a serine protease, tPA is inhibited primarily by its serpin plasminogen activator inhibitor 1 (PAI-1) (10). In obesity, plasminogen activator activity and fibrinolysis in blood are reduced due to an increase in PAI-1 (9, 14). Coincidentally, tPA is also increased in obesity (15, 16), which may represent a compensatory response. These observations raise a number of critical unanswered questions, namely, what are the sources and regulatory mechanisms controlling PAI-1 and tPA in obesity, and does the tPA response partially compensate for the impairment in fibrinolysis caused by high PAI-1 in obesity?

One source of PAI-1 is adipocytes, and elevated adipocyte PAI-1 has been proposed as the dominant source for increased PAI-1 and the consequent fibrinolysis defect in obesity (9, 14, 15). Arguing against this idea is the observation that when blood from subcutaneous adipose arteries and veins was assayed for PAI-1 protein and activity in obese humans, there was no PAI-1 arteriovenous gradient (17). These data raise the possibility that one or more other sources of circulating PAI-1 may be important in obesity. Hepatocytes are sensitive to the metabolic stress that occurs in obesity and, as is the case with tPA (13), hepatocytes can produce PAI-1 (18–21). However, the regulation of hepatocyte PAI-1 in obesity and whether an increase in hepatocyte-derived PAI-1 contributes to the fibrinolysis defect in obesity are not known. These gaps of knowledge also exist for hepatocyte tPA in obesity. In this context, we now show that hepatocytes are a substantial source of both plasma tPA and PAI-1 in mice with diet-induced obesity. The increase in hepatocyte PAI-1 in obesity is linked to a decrease in the PAI-1 (*SERPINE1*) gene corepressor Rev-Erbα. Moreover, reducing hepatocyte PAI-1 in obese mice blocked the increase in both liver tPA and functionally active plasma tPA, indicating the existence of a hepatocyte PAI-1/tPA pathway in obesity. Although this tPA response pathway is partially counteracted by a coexisting tPA-suppressive pathway mediated by the corepressor DACH1, we provide evidence that the PAI-1/tPA pathway limits the degree of impairment of fibrinolysis in obesity. Accordingly, the fibrinolysis defect in obesity becomes even worse when the PAI-1/tPA pathway is genetically targeted in mice. The mechanism of the PAI-1/tPA pathway involves induction of the tPA gene *PLAT* through a pathway in which PAI-1, through its receptor LRP1, activates the *PLAT* inducer cAMP-responsive element-binding protein 1 (CREB1). The PAI-1/*PLAT* pathway is present in primary human hepatocytes, and livers, but not adipose tissue, from obese and lean humans showed associations among DACH1, tPA, and *PLAT* that are consistent with these findings. These findings add insight into the regulation of fibrinolysis in obesity, thereby suggesting new strategies for lowering thrombotic risk in this condition.

## Results

### Hepatocyte tPA is increased in diet-induced obese mice, but a larger increase in hepatocyte PAI-1 causes a net impairment of fibrinolysis.

Plasma tPA protein concentration was increased in obese versus lean mice, but plasma tPA activity was reduced, plasma clot-lysis time was delayed, and time to occlusive carotid arterial thrombosis induced by photochemical injury was shortened in obese mice (Supplemental Figure 1A; supplemental material available online with this article; https://doi.org/10.1172/JCI135919DS1). These findings are consistent with data in obese humans (22–25). Based on previous reports (16, 24, 26, 27), we reasoned that reduced plasma tPA activity despite increased tPA protein could be explained by increased plasma PAI-1. Consistent with this formulation, plasma PAI-1 was markedly increased in the plasma of obese mice (Supplemental Figure 1B).

**Figure 1:**
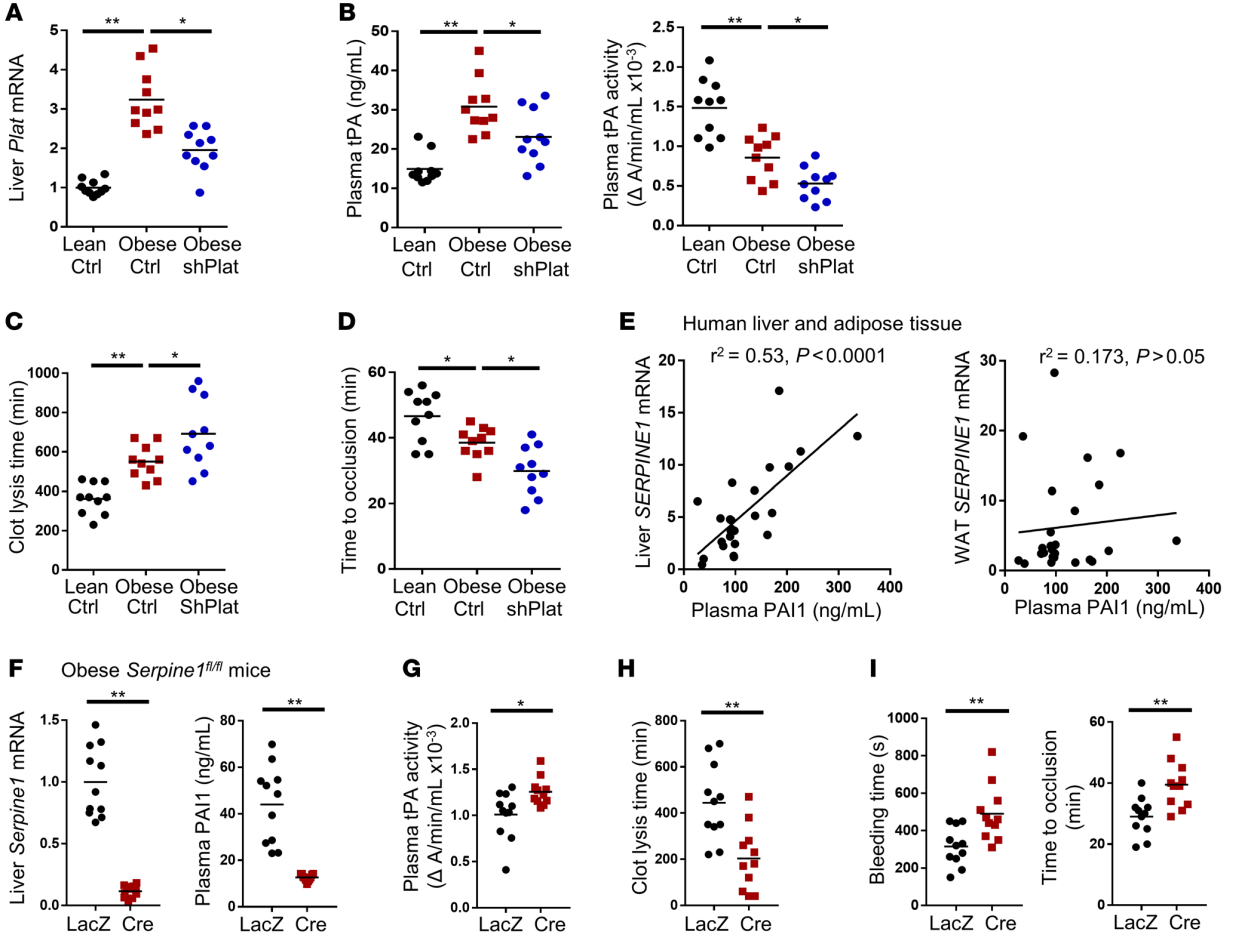
Hepatocyte tPA is increased in DIO mice, but a larger increase in hepatocyte PAI-1 causes a net impairment of fibrinolysis. (**A**–**C**) Chow-fed mice (Lean) or DIO mice were injected intravenously with AAV8-H1-shPlat (shPlat) to silence the tPA gene *Plat* in hepatocytes or with control AAV8-H1-Scr (Ctrl). After 4 weeks, the following parameters were measured: (**A**) liver *Plat* mRNA; (**B**) plasma tPA protein concentration and plasma tPA activity; (**C**) fibrinolytic activity measured by euglobulin clot-lysis assay; and (**D**) time to occlusive carotid arterial thrombosis induced by rose bengal/laser photochemical injury. Horizontal lines in the dot-density plots indicate mean values. *n* = 10 mice/group. *P < 0.05; **P < 0.01, 1-way ANOVA followed by Tukey’s test. (**E**) Samples of human liver and omental white adipose tissue (WAT) from the subjects described in Supplemental Table 1 were assayed for *SERPINE1* mRNA, and the plasma from these subjects was assayed for PAI-1 concentration. The graph shows plots of the indicated correlations, which were analyzed by linear regression, with the *r^2^* and *P* values indicated in the graph. (**F**–**I**) *Serpine1^fl/fl^* mice were fed a high-fat diet for 4 months and then injected intravenously with AAV8-TBG-Cre (Cre) to target the PAI-1 gene *Seprine1* in hepatocytes or with control AAV8-TBG-LacZ (LacZ). After 4 weeks, the following parameters were measured: (**F**) liver *Serpine1* mRNA and plasma PAI-1 protein concentration; (**G**) plasma tPA activity measured by enzymatic assay; (**H**) plasma fibrinolytic activity measured by the euglobulin clot-lysis assay; and (**I**) tail-bleeding time and time to occlusive carotid arterial thrombosis induced by rose bengal/laser photochemical injury. *n* = 11 mice/group. *P < 0.05; **P < 0.01, 2-tailed Student’s *t* test.

To elucidate the cellular source of increased tPA and PAI-1 expression in obesity, we focused on hepatocytes because hepatocytes are profoundly affected by obesity and, in lean mice, hepatocytes contribute substantially to the basal level of plasma tPA and to fibrinolysis in response to vessel injury (13). Beginning with tPA, we found that *Plat* mRNA was increased in the livers of obese versus lean mice, although tPA activity in the liver was lower (Supplemental Figure 1C). Consistent with our previous observation (13), hepatic and endothelial *Plat* mRNA levels were comparable in lean mice, but only hepatic *Plat* mRNA was increased by obesity (Supplemental Figure 1D). To test the functional significance of the increase in liver tPA in obesity, we silenced hepatocyte tPA in obese mice using hepatocyte-specific shRNA (AAV8-H1-shPlat) (13), titrating the dosage of the virus to lower hepatic *Plat* close to the level in lean mice, but not lower (Figure 1A). This level of silencing (~40%) led to an approximately 25% decrease in plasma tPA protein, an approximately 32% decrease in plasma tPA activity, an approximately 26% increase in plasma clot-lysis time, and an approximately 22% decrease in postinjury carotid artery–occlusion time (Figure 1, B–D), all without changing plasma levels of PAI-1 protein and α2-antiplasmin (A2AP), the major inhibitor of plasmin (Supplemental Figure 1, E and F). These data demonstrate the functional significance of increased hepatocyte tPA in obesity, i.e., the defect in fibrinolysis in obesity is even greater when this increase is prevented. Thus, the obesity-induced increase in hepatocyte tPA limits the fibrinolytic impairment in obesity.

We next turned our attention to PAI-1 and its mRNA, *Serpine1*. We found markedly increased expression of *Serpine1* mRNA in the livers of obese versus lean mice (Supplemental Figure 1G). Although PAI-1 protein levels were increased in both liver and omental white adipose tissue in obese versus lean mice, liver PAI-1 protein was higher than adipose PAI-1 protein (Supplemental Figure 1H). In human specimens, plasma PAI-1 protein was strongly correlated with liver *SERPINE1* mRNA level, but not with *SERPINE1* mRNA, in adipose tissue (Figure 1E and Supplemental Table 1). Most importantly, specific targeting of hepatocyte PAI-1 in obese *Serpine1^fl/fl^* mice (28) using AAV8-TBG-Cre, which silences floxed genes exclusively in hepatocytes (13, 29–32), decreased plasma PAI-1 protein approximately 70%, increased plasma tPA activity, shortened clot-lysis time, and increased both tail-bleeding time and time to occlusive thrombosis (Figure 1, F–I), all without changing plasma A2AP levels (Supplemental Figure 1I). In summary, obesity is associated with increased expression of tPA and PAI-1 in hepatocytes, both of which influence plasma fibrinolysis. Although the dominant effect is impaired fibrinolysis secondary to increased hepatocyte PAI-1, the increase in hepatocyte tPA limits the degree of this impairment.

### Increased hepatocyte DACH1 in obesity limits the rise in tPA and thereby contributes to impaired fibrinolysis.

**Figure 2:**
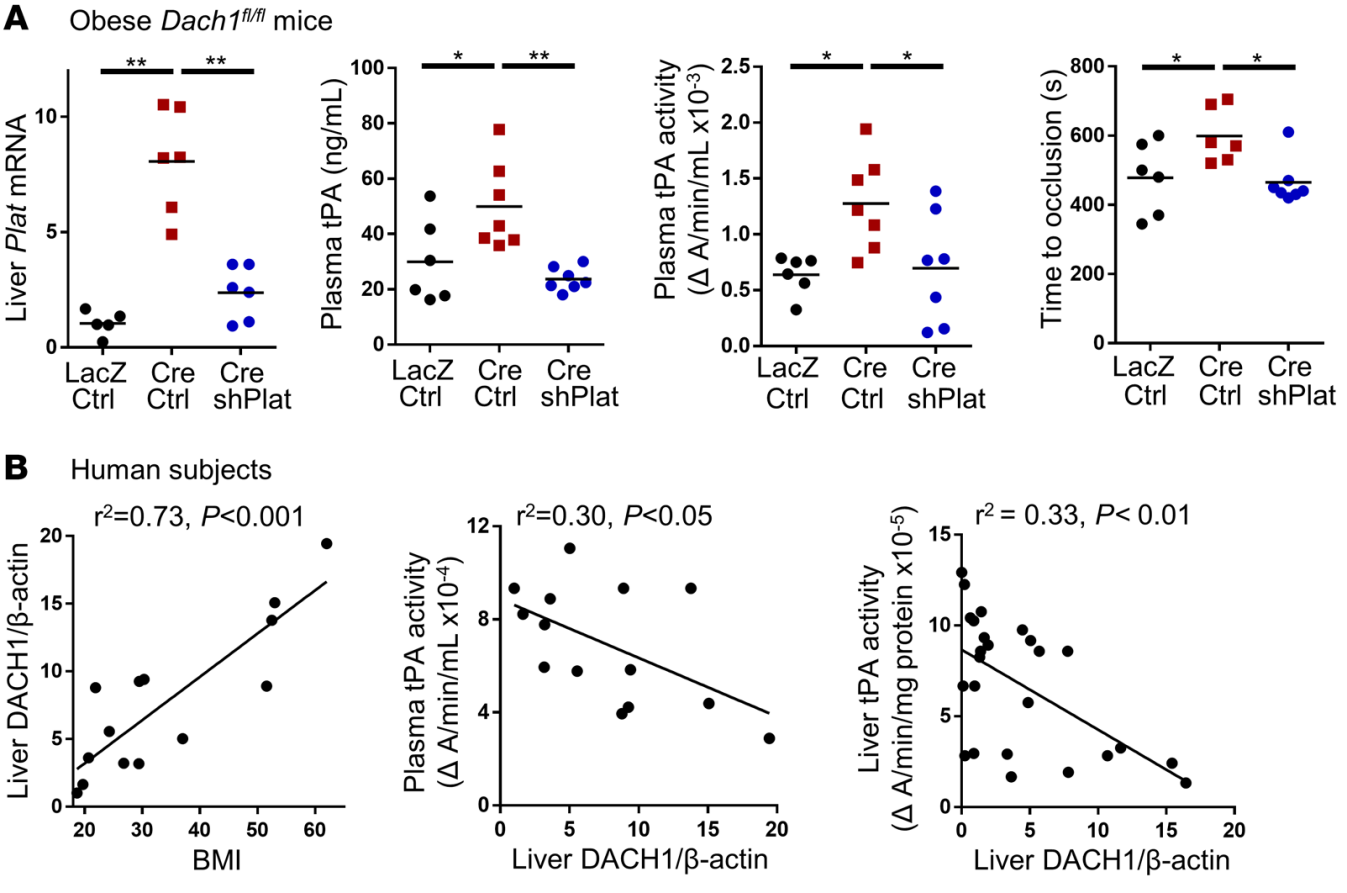
Increased hepatocyte DACH1 in obesity limits the rise in tPA and thereby contributes to impaired fibrinolysis. (**A**) *Dach1^fl/fl^* mice were fed a high-fat diet for 4 months and then injected intravenously with AAV8-TBG-Cre to target the *Plat* corepressor *Dach1* in hepatocytes or with control AAV8-TBG-LacZ. Mice were also injected with AAV8 control virus or AAV8-H1-shPlat, as indicated. After 4 weeks, the following parameters were measured: liver *Plat* mRNA, plasma tPA protein concentration, plasma tPA activity, and time to occlusive carotid arterial thrombosis induced by rose bengal/laser photochemical injury. Horizontal lines in the dot-density plots indicate mean values. *n* = 5–7 mice/group. *P < 0.05; **P < 0.01, 1-way ANOVA followed by Tukey’s test. (**B**) For the left and middle graphs, liver specimens from 14 human subjects with a wide range of BMI, as described previously (22), were assayed for DACH1/β-actin densitometric ratio on immunoblot, as shown in Supplemental Figure 3B, and samples of their plasma were assayed for tPA activity. For the right graph, liver specimens from 25 human subjects, as previously described (13), were assayed for liver DACH1/β-actin ratio (13) and tPA activity. Graphs show plots of the indicated correlations, which were analyzed by linear regression, with *r^2^* and *P* values indicated in the graph.

The expression of hepatocyte *PLAT* is negatively regulated in lean mice by the corepressor DACH1, which represses the *PLAT* inducer ATF6 (13). We reasoned that this pathway would be particularly relevant to obesity, as hepatocyte DACH1 is increased in the livers of obese mice and humans (32), which we verified here (Supplemental Figure 2A). To test this idea, we injected diet-induced obese (DIO) *Dach1^fl/fl^* mice with AAV8-TBG-Cre, which targets DACH1 exclusively in hepatocytes (13), or with control AAV8-TBG-LacZ (Supplemental Figure 2B). Consistent with the hypothesis, targeting hepatocyte DACH1 (HC-DACH1–KO mice) increased liver *Plat* mRNA, plasma tPA concentration and activity, and time to occlusive carotid thrombosis (Figure 2A). To determine whether the increased fibrinolytic activity was due to increased hepatic tPA expression, we silenced hepatocyte *Plat* in the HC-DACH1–KO mice using AAV8-H1-sh-tPA to the level seen in control AAV8-TBG-LacZ–treated obese mice. This intervention normalized all of the fibrinolytic parameters in the obese HC-DACH1–KO mice (Figure 2A). Neither deleting hepatocyte DACH1 nor treating these mice with shPlat changed plasma PAI-1 concentration (Supplemental Figure 2C). Finally, we looked for correlations among BMI, liver DACH1, liver tPA activity, and plasma tPA activity in humans. Liver DACH1 levels rose with increasing BMI, as shown in our previous study (13) and quantified here, and tPA activities in liver and plasma were negatively correlated with liver DACH1 (Figure 2B and Supplemental Table 2). Similar correlations were observed in mice with a wide range in body weight (Supplemental Figure 2D). In summary, the increase in hepatocyte tPA in obesity is limited by the counteracting process of DACH1-mediated *Plat* repression, and the resulting net level of hepatic tPA expression is not high enough to overcome PAI-1–mediated impaired fibrinolysis.

### The increase in hepatocyte PAI-1 drives the increase in hepatocyte tPA in obesity.

**Figure 3:**
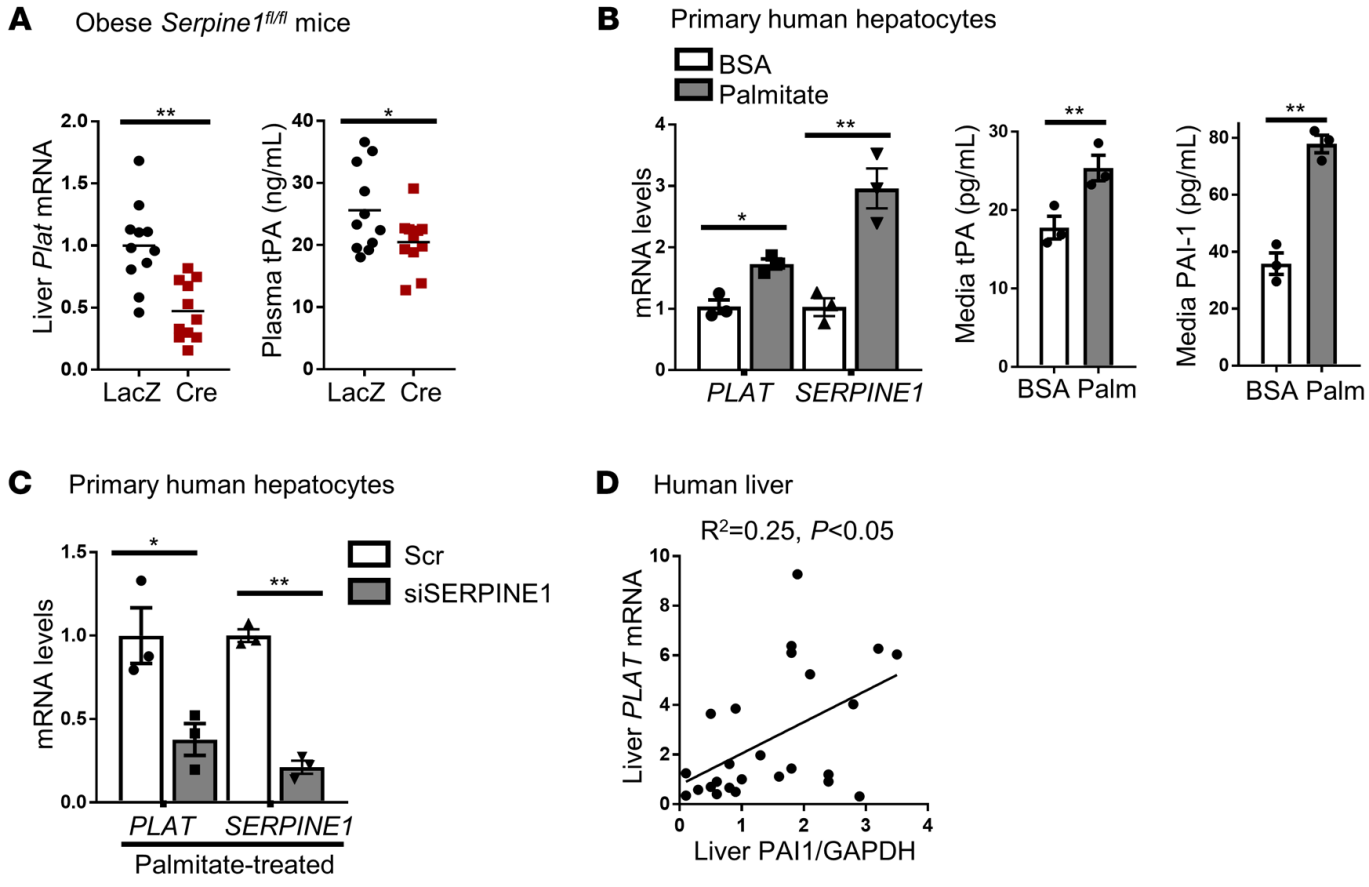
The increase in hepatocyte PAI-1 drives the increase in hepatocyte tPA in obesity. (**A**) *Serpine1^fl/fl^* mice were fed a high-fat diet for 4 months and then injected intravenously with either AAV8-TBG-Cre or control AAV8-TBG-LacZ. After 4 weeks, liver *Plat* mRNA and plasma tPA protein concentrations were measured. Horizontal lines in the dot-density plots indicate mean values. *n* = 11 mice/group. *P < 0.05; **P < 0.01, 2-tailed Student’s *t* test. (**B**) Human primary hepatocytes were treated for 16 hours with 100 μM palmitate (PALM) in BSA solution, or BSA solution control. The cells were assayed for *PLAT* and *SERPINE1* mRNA, and the media were assayed for tPA and PAI-1 protein concentration. (**C**) Human primary hepatocytes were transfected with siSERPINE1 or scrambled control (Scr). After 24 hours, cells were treated with 100 μM palmitate for an additional 16 hours and then assayed for *PLAT* and *SERPINE1* mRNA. In **B** and **C**, results are shown as mean ± SEM. *n* = 3 sets of cells/group. *P < 0.05; **P < 0.01, 2-tailed Student’s *t* test. (**D**) Liver specimens from the subjects described in Supplemental Table 1 were assayed for PAI‑1/GAPDH densitometric ratio by immunoblot, as shown in Supplemental Figure 3C, and for *PLAT* mRNA. These data were then subjected to correlation analysis, as described in the legend for Figure 2B.

Having elucidated a factor that lowers hepatocyte tPA in obesity, i.e., the increase in DACH1, we next sought to understand the counteracting mechanism that causes the net increase in hepatocyte and plasma tPA in obesity. In this context, we noted that liver *Plat* mRNA and plasma tPA protein were lower in HC–PAI-1–KO than in control obese mice (Figure 3A). These data raised the possibility that PAI-1 induces tPA in hepatocytes in obesity, perhaps representing a compensatory response. We tested this possibility in an in vitro hepatocyte model by incubating primary human hepatocytes with palmitate, which we and others have found can recapitulate certain aspects of hepatocyte biology seen in obesity (32, 33). This treatment increased both *PLAT* and *SERPINE1* mRNAs and protein in culture medium (Figure 3B), thus mimicking the response in livers and plasma of obese mice. Most importantly, silencing *SERPINE1* in these cells markedly lowered hepatocyte *PLAT* mRNA (Figure 3C). We obtained similar results with primary mouse hepatocytes (Supplemental Figure 3, A and B). Next, we examined PAI-1 protein and *PLAT* mRNA in 25 human liver specimens that had a wide range of PAI-1 protein expression (Supplemental Figure 3C and Supplemental Table 2). The data show a statistically significant correlation between PAI-1 and *PLAT* in the livers of these subjects (Figure 3D), consistent with the findings in mice and primary human hepatocytes. Together, these data provide evidence for a cell-autonomous PAI-1/*PLAT* pathway in hepatocytes.

**Figure 4:**
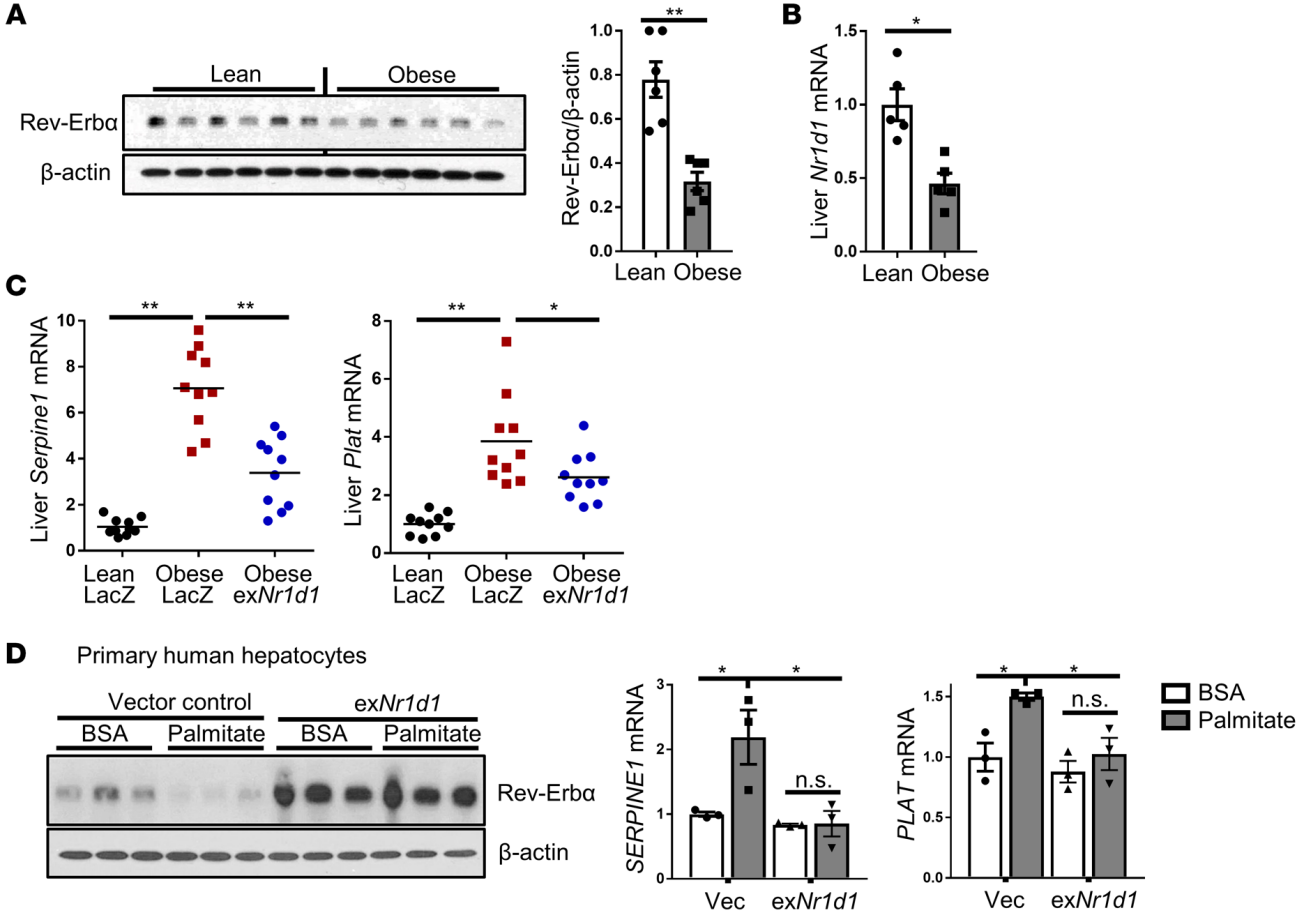
Decreased Rev-Erbα in the livers of obese mice and in palmitate-treated primary human hepatocytes elevates both PAI-1 and tPA expression. (**A** and **B**) Livers of lean and DIO mice were assayed for Rev-Erbα protein and β-actin loading control by immunoblot, with densitometric quantification shown, and for *Nr1d1* mRNA. *n* = 5–6 mice/group. Data are represented as mean ± SEM. *P < 0.05; **P < 0.01, 2-tailed Student’s *t* test. (**C**) Lean and obese mice were injected intravenously with AAV8-TBG-Nr1d1 to increase Rev-Erbα expression in hepatocytes or with AAV8-TBG-LacZ control, as indicated. After 4 weeks, liver *Serpine1* and *Plat* mRNA levels were measured. Horizontal lines in dot-density plots indicate mean values. *n* = 10 mice/group. *P < 0.05; **P < 0.01, 1-way ANOVA followed by Tukey’s test. (**D**) Human primary hepatocytes were transfected with a plasmid expressing Nr1d1 to increase expression of Rev-Erbα; transfection with empty vector (Vec) served as the control. After 24 hours, cells were treated with 100 μM palmitate for 16 hours and then assayed for Rev-Erbα protein by immunoblot and for *SERPINE1* and *PLAT* mRNA by quantitative PCR (qPCR). *n* = 3 sets of cells/group. Data are represented as mean ± SEM. *P < 0.05, 1-way ANOVA followed by Tukey’s test.

To seek further evidence for this pathway in obesity, we used an alternative strategy to limit endogenous hepatic PAI-1 expression. Rev-Erbα is a transcriptional repressor of PAI-1, and it was shown to be lower in adipose tissue of obese mice than in lean mice (34, 35). We found that Rev-Erbα protein and mRNA (*Nr1d1*) were lower in the livers of obese versus lean mice (Figure 4, A and B). Accordingly, we asked whether restoring Rev-Erbα in hepatocytes in obese mice would lower PAI-1 and thereby lower *Plat* expression via the new pathway revealed here. For this purpose, obese mice were injected with AAV8-TBG-Nr1d1, which increased liver *Nr1d1* and Rev-Erbα to levels closer to those of lean mice (Supplemental Figure 4A), or control AAV8-TBG-LacZ. As predicted from prior reports, increasing hepatocyte Rev-Erbα in obese mice lowered liver *Serpine1*, and most importantly, it also lowered liver *Plat* (Figure 4C). Consistent with hepatocytes being an important source of circulating PAI-1 and with the dominant effect of hepatocyte PAI-1 on fibrinolysis, the net effect of hepatocyte Rev-Erbα restoration in obese mice was lower plasma PAI-1, increased plasma tPA activity, and shorter time to clot lysis (Supplemental Figure 4B). These links among Rev-Erbα, *SERPINE1*, and *PLAT* are cell autonomous, as demonstrated with palmitate-treated primary human hepatocytes: palmitate lowered Rev-Erbα, and restoration of Rev-Erbα in these cells lowered both *SERPINE1* and *PLAT* mRNA (Figure 4D). Further, silencing Rev-Erbα in primary human hepatocytes using siNR1D1 increased *PLAT*, and this effect was dependent on the ability of siNR1D1 to increase PAI-1, as simultaneous silencing of PAI-1 using siSERPINE1 prevented the *PLAT*-raising effect of siNR1D1 (Supplemental Figure 4C). These combined data reveal the presence of a hepatocyte PAI-1/*Plat* pathway in obesity. Although the effect of PAI-1 is dominant over that of tPA, the presence of this pathway suggests that fibrinolysis would be even more impaired in obesity if this pathway were not present.

**Figure 5:**
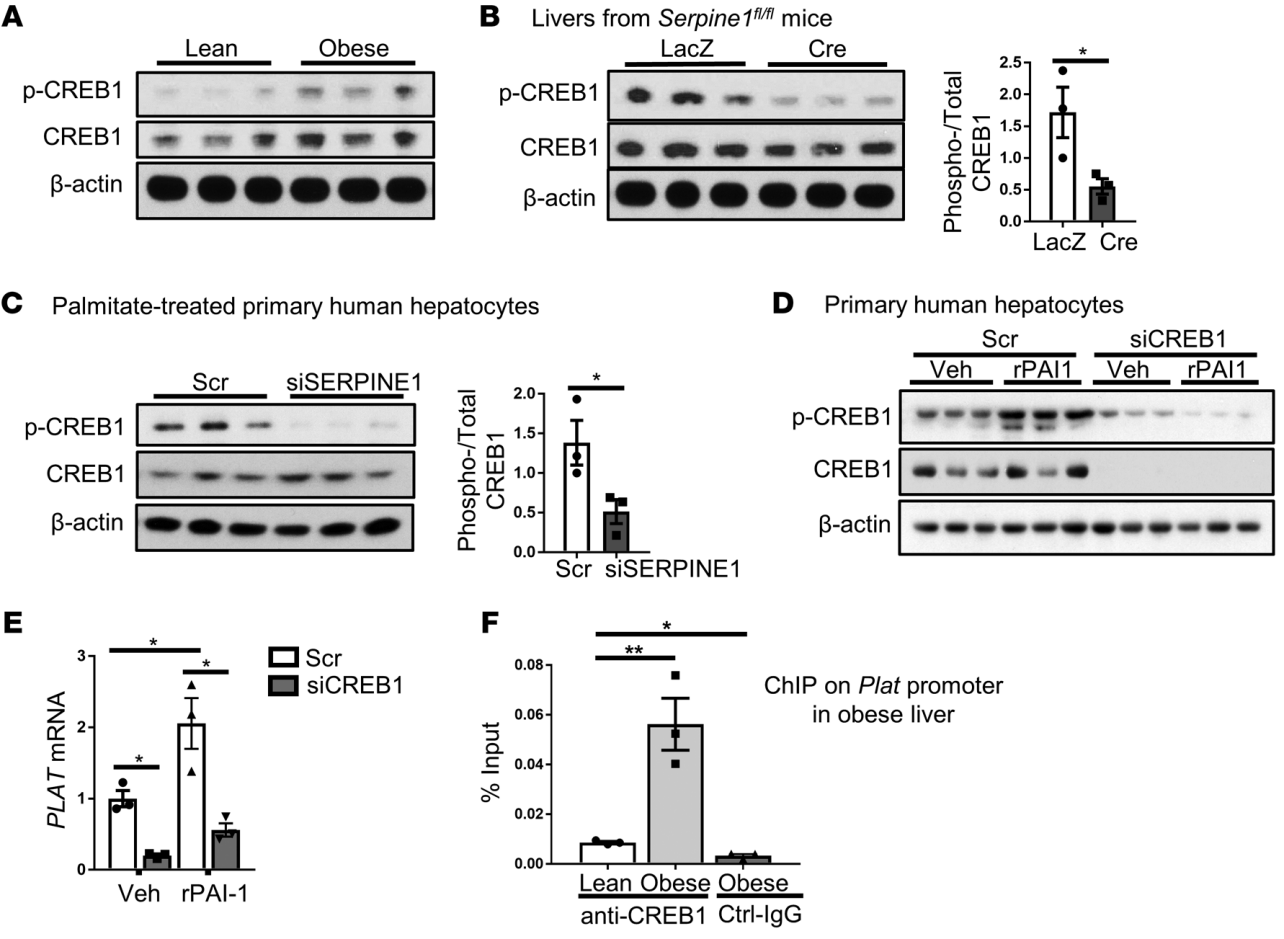
PAI-1 induces CREB1 phosphorylation, which then induces tPA expression in hepatocytes. (**A**) Livers of lean and obese mice were assayed for p-CREB1 and total CREB1 by immunoblot (*n* = 3 mice/group). (**B**) *Serpine1^fl/fl^* mice were fed a high-fat diet for 4 months and then injected intravenously with either AAV8-TBG-Cre or control AAV8-TBG-LacZ. After 4 weeks, livers were assayed for p-CREB1 and total CREB1 by immunoblot, with densitometric quantification shown. Data are shown as mean ± SEM. *P < 0.05, 2-tailed Student’s *t* test. (**C**) Human primary hepatocytes were transfected with siSERPINE1 or scrambled control. After 24 hours, cells were treated with 100 μM palmitate for 16 hours, followed by assay of phosphorylated and total CREB1 by immunoblot, with densitometric quantification shown. Data are represented as mean ± SEM. *P < 0.05, 2-tailed Student’s *t* test. (**D** and **E**) Human primary hepatocytes were transfected with siCREB1 or scrambled control. After 24 hours, cells were treated for 8 hours with 1 μg rPAI-1/mL culture medium or vehicle control (Veh). Cells were then assayed for phosphorylated and total CREB1 by immunoblot and for *PLAT* mRNA by qPCR. *n* = 3 sets of cells/group. Data are represented as mean ± SEM. *P < 0.05, 1-way ANOVA followed by Tukey’s test. (**F**) Nuclear extracts from the livers of lean or obese mice were subjected to ChIP assay using anti-CREB1 or control IgG (Ctrl-IgG). The proximal promoter region containing the CREB1-binding sequence in the *Plat* gene was amplified by qPCR and normalized to the values obtained from input DNA. *n* = 3 mice/group. Data are represented as mean ± SEM. *P < 0.05; **P < 0.01, 1-way ANOVA followed by Tukey’s test.

*PAI-1 induces hepatic tPA expression through a LRP1*/*p-CREB1 pathway*. To explore the mechanism whereby PAI-1 induces tPA, we first considered the possibility that PAI-1 reduces DACH1. However, liver DACH1 protein was not affected by deletion of hepatocyte PAI-1 (Supplemental Figure 5A). We next considered the possibility that PAI-1 increases CREB1, which is a transcriptional activator of the *PLAT* gene in human endothelial cells (36). Although the role of CREB1 in *PLAT* expression in hepatocytes is not known, CREB1 is expressed in hepatocytes (37). We first compared the livers of lean and obese mice and found that both total and activated (phosphorylated) CREB1 (p-CREB1) were increased in obesity (Figure 5A). Next, we showed that genetic deletion or silencing of hepatocyte PAI-1 decreased p-CREB1 in the livers of obese mice (Figure 5B) and in palmitate-treated primary human hepatocytes (Figure 5C). To further establish that PAI-1 activates CREB1 in hepatocytes, we showed that incubation of primary human hepatocytes with recombinant PAI-1 (rPAI-1) increased p-CREB1 (Figure 5D). Most importantly, rPAI-1 increased *PLAT* expression in these cells, and we found that both basal PLAT and rPAI-1–induced PLAT were inhibited by silencing CREB1 (Figure 5E and Supplemental Figure 5B). We obtained similar results using primary mouse hepatocytes (Supplemental Figure 5C). As predicted, ChIP assays in mouse liver revealed a 5-fold enrichment of CREB1 at the consensus site in the *Plat* gene promoter in obese versus lean liver, but not in the nonspecific *Rplp0* promoter (Figure 5F and Supplemental Figure 5D). These data support the existence of a PAI-1/CREB1/*PLAT* pathway in hepatocytes that, owing to the increase in hepatocyte PAI-1 in obesity, is activated in obese liver.

**Figure 6:**
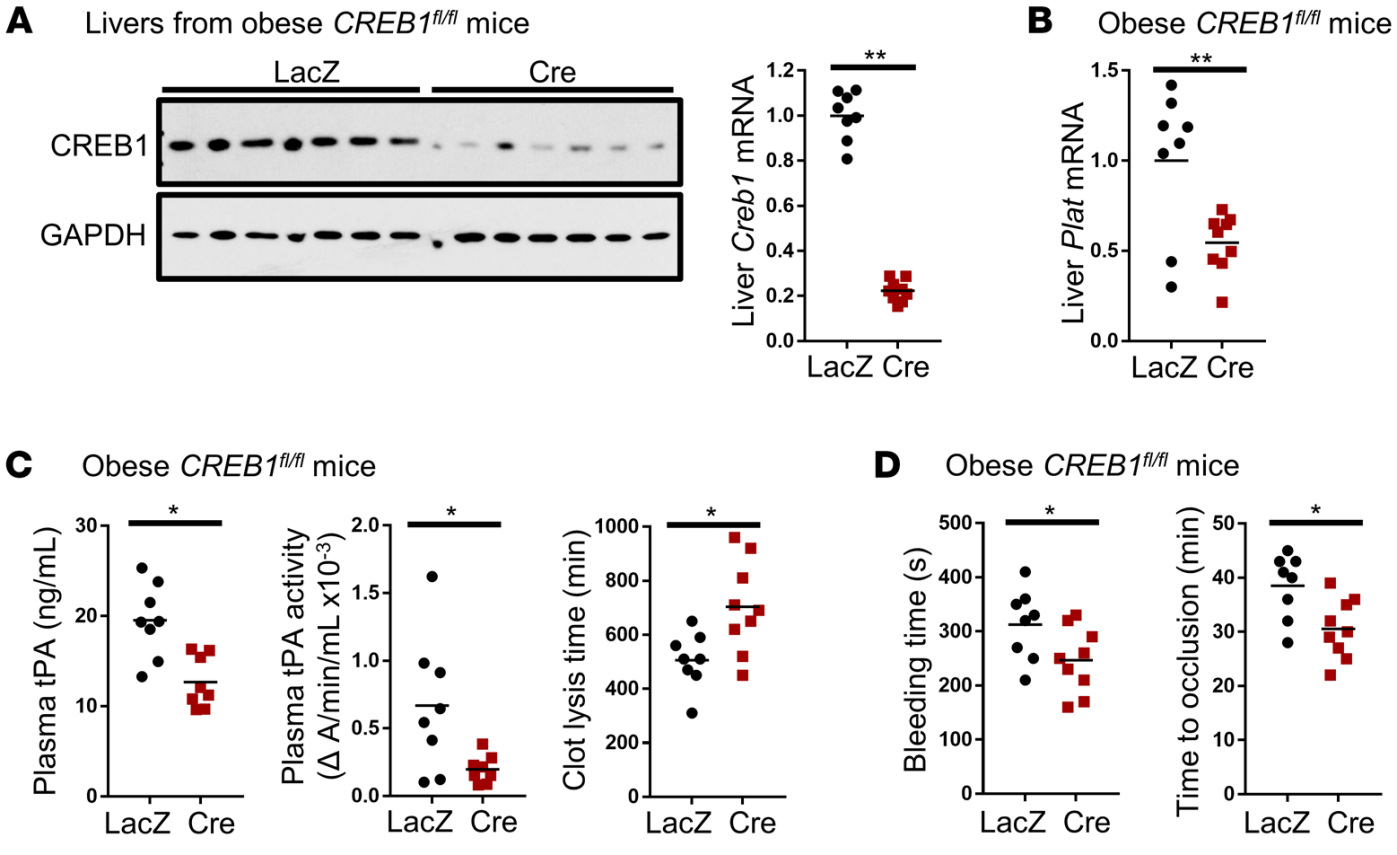
Obese mice with hepatocyte CREB1 deficiency have decreased plasma tPA protein concentration and activity, delayed clot-lysis time, and shortened time to arterial thrombotic occlusion. *Creb1^fl/fl^* mice were fed a high-fat diet for 4 months and then injected intravenously with AAV8-TBG-Cre to target *Creb1* in hepatocytes or with control AAV8-TBG-LacZ. After 4 weeks, the following parameters were assayed: (**A** and **B**) liver CREB1 by immunoblot and liver *Creb1* and *Plat* mRNA by qPCR; (**C**) plasma tPA protein concentration, plasma tPA activity, and plasma fibrinolytic activity measured by the euglobulin clot-lysis assay; (**D**) tail-bleeding time and time to occlusive carotid arterial thrombosis induced by rose bengal/laser photochemical injury. *n* = 7–9 mice/group. *P < 0.05; **P < 0.01, 2-tailed Student’s *t* test.

To examine the in vivo relevance of this pathway, we silenced hepatic CREB1 in obese mice by treating DIO CREB1*^fl/fl^* mice with AAV8-TBG-Cre (HC-CREB1–KO) (Figure 6A). Consistent with the hypothesized pathway, targeting hepatocyte CREB1 lowered liver *Plat* mRNA (Figure 6B). Importantly, this decrease in liver *Plat* mRNA was associated with decreased plasma tPA protein and plasma tPA activity, increased clot-lysis time, and decreased time to occlusion after carotid injury (Figure 6, C and D), all without a change in plasma PAI-1 (Supplemental Figure 5E).

**Figure 7:**
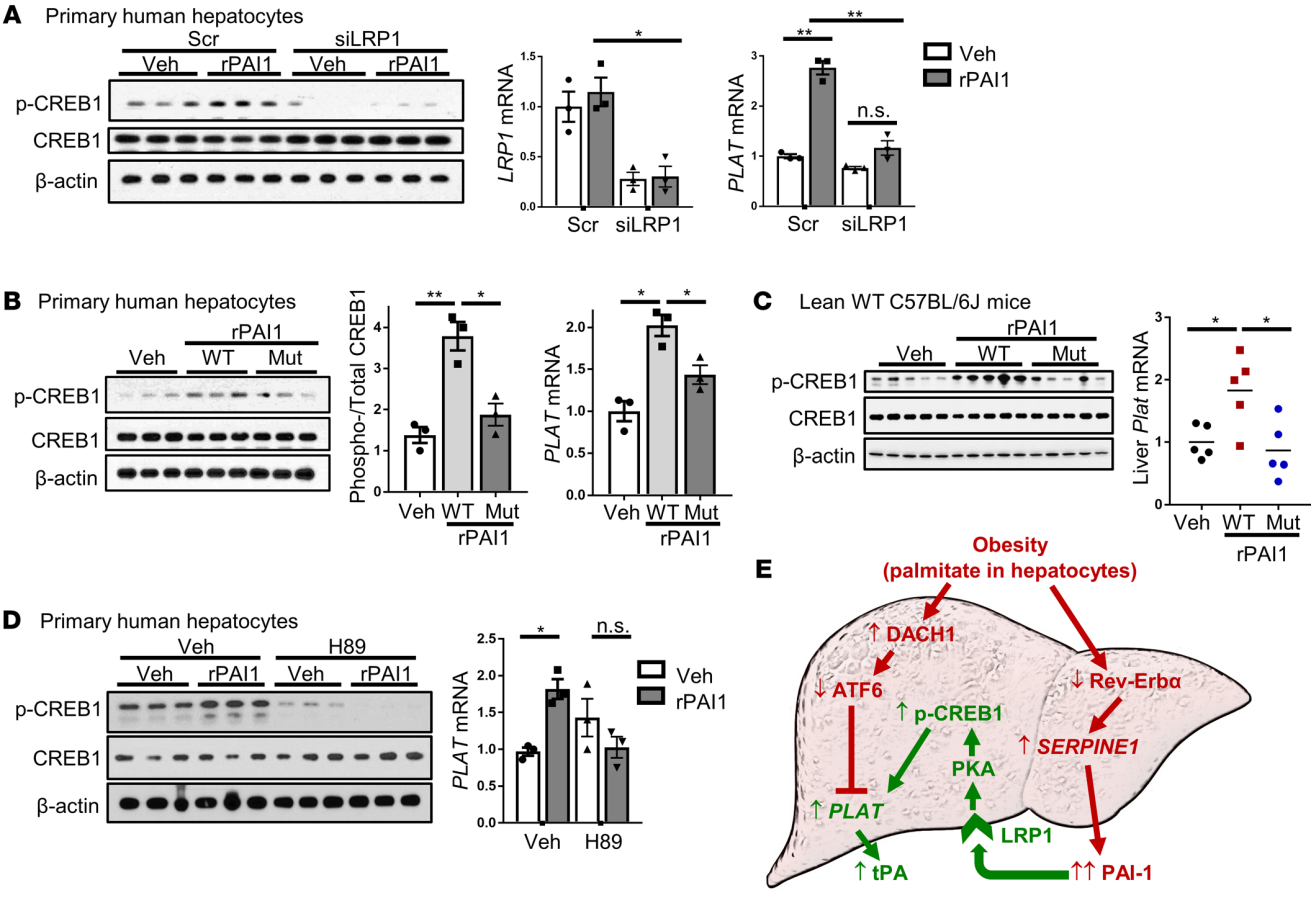
PAI-1 induces hepatic tPA expression through an LRP1/p-CREB1/PKA pathway. (**A**) Human primary hepatocytes transfected with si-LRP1 or scrambled control were treated for 8 hours with 1 μg rPAI-1/mL culture medium and then assayed for phosphorylated and total CREB1 and *LRP1* and *PLAT* mRNA. (**B**) Human primary hepatocytes treated for 8 hours with 1 μg/mL WT rPAI-1 (WT) or a mutant rPAI-1 (Mut) with enzymatic activity, but lacking the LRP1-interacting heparin-binding domain, were assayed for p-CREB1 and total CREB1 and *PLAT* mRNA. For **A** and **B**, results are represented as mean ± SEM. *n* = 3 sets of cells/group. *P < 0.05; **P < 0.01, 1-way ANOVA followed by Tukey’s test. (**C**) Lean mice were injected intravenously with 100 μg WT or mutant rPAI-1 or vehicle control. After 90 minutes, livers were assayed for p-CREB1 and total CREB1 and *Plat* mRNA. *n* = 5 mice/group. *P < 0.05, 1-way ANOVA followed by Tukey’s test. (**D**) Human primary hepatocytes were treated for 4 hours with 10 μM of PKA inhibitor H89 and then treated 4 hours with 1 μg rPAI-1/mL. Cells were then assayed for p-CREB1 and total CREB1 and *PLAT* mRNA. *n* = 3 sets of cells/group. *P < 0.05, 1-way ANOVA followed by Tukey’s test. (**E**) Summary schematic. Red font and arrows depict pathways that contribute to impaired fibrinolysis in obesity, and pathway in green depicts the compensatory PAI/tPA pathway. The 2 pathways that contribute to impaired fibrinolysis are increased *SERPINE1* due to decreased Rev-Erbα and partial suppression of *PLAT* due to increased DACH1, which represses the *PLAT* inducer ATF6. The compensatory pathway is mediated by an increase in the *PLAT* inducer p-CREB1 downstream of a PAI-1/LRP1/PKA signaling cascade. See text for details.

Ligation of LDL receptor-related protein 1 (LRP1), the major cellular receptor for PAI-1, can increase intracellular cAMP levels and the activity of protein kinase A (PKA) (38), the kinase that phosphorylates and activates CREB1 (39). Further, LRP1 has been shown to stimulate CREB1 transcription activity in neurons (40) and adipocytes (41). As LRP1 is abundant on the surface of hepatocytes, we hypothesized that PAI-1 activates CREB1 through LRP1-PKA–mediated signaling to increase tPA expression in obesity. Consistent with this idea, silencing LRP1 in primary human hepatocytes suppressed the rPAI-1–mediated increases in p-CREB1 and *PLAT* mRNA (Figure 7A). Further, treating these cells with a mutant form of rPAI-1 lacking the LRP1-interacting heparin-binding domain markedly reduced CREB1 phosphorylation and *PLAT* expression compared with WT rPAI-1 (Figure 7B). We then tested this point in vivo by injecting lean mice with WT rPAI-1, which we predicted would “mimic” the pathway seen in obese liver, or with LRP1-binding mutant rPAI-1, which should be inactive. The total plasma PAI-1 concentration in both WT- and mutant rPAI-1–injected mice reached levels similar to those seen in obese mice (Supplemental Figure 5F compared with Supplemental Figure 1B). WT rPAI-1 increased p-CREB and liver *Plat*, whereas mutant rPAI-1 did not (Figure 7C). With regard to the role of PKA (above), we found that rPAI-1 induction of p-CREB and *PLAT* in primary human hepatocytes was prevented by treating the cells with the PKA inhibitor H89 (Figure 7D). Together, these data provide support for a pathway in which the increase in hepatocyte PAI-1 in obesity, mediated at least in part by suppression of Rev-Erbα, activates an LRP1/PKA/p-CREB/*PLAT* pathway that lessens the magnitude of the PAI-1–mediated decrease in fibrinolysis in obesity. Figure 7E summarizes this PAI-1/LRP1/PKA/p-CREB1/*PLAT* pathway, as integrated with the DACH1 and Rev-Erbα pathways.

## Discussion

Obesity has reached epidemic proportions worldwide and is a major contributor to a number of widespread chronic diseases, notably type 2 diabetes, cardiovascular disease, and nonalcoholic steatohepatitis (NASH) (42, 43). Among the more serious consequences of obesity, and one that contributes to cardiovascular disease, is increased risk of thrombosis (1, 3–5, 8, 44), which causes 1 in 4 deaths worldwide (45). Impaired fibrinolysis is a significant contributor to obesity-associated thrombosis (8, 14, 46–50), but the underlying molecular mechanisms linking obesity to defects in fibrinolysis have remained largely unknown. Two critical gaps in this area of research are how obesity creates an imbalance between PAI-1 and tPA, the 2 major regulators of fibrinolysis, and the cellular source or sources of PAI-1 and tPA that contribute to this imbalance. A related gap is the mechanism and functional significance of a seemingly paradoxical observation that plasma tPA protein levels are actually increased in obese humans (15, 16). In this context, the major conclusions of this study are as follows: (a) hepatocytes are an important source for the increases in plasma PAI-1 and tPA in obesity; (b) hepatocyte-derived PAI-1 contributes substantially to impaired fibrinolysis in obesity; (c) hepatocyte tPA (*PLAT*) gene expression in obesity is regulated negatively by DACH1 and positively by PAI-1, which may represent a “compensatory” feedback pathway; (d) the net result of these opposing modes of hepatocyte *PLAT* regulation in obesity is an increase in plasma tPA protein, mimicking the findings in humans; and (e) although the PAI-1–mediated impairment of fibrinolysis in obesity is dominant, this impairment would be even worse without the hepatocyte PAI-1/*PLAT* pathway.

Previous studies have identified adipose tissue as a major determinant of circulating PAI-1 in obesity based on PAI-1 mRNA (*SERPINE1*) and protein levels among various tissues (9, 14, 15). However, another report showed that there was no arteriovenous gradient for PAI-1 protein and activity in adipose tissue in obese humans (17). Additionally, plasma PAI-1 was more closely correlated to *Serpine1* mRNA in liver than in adipose tissue in obese mice (51), consistent with our findings in humans (Figure 1E and Supplemental Figure 1H). In our study, obesity in mice increased plasma tPA by approximately 3-fold and plasma PAI-1 by approximately 9-fold (Supplemental Figure 1, A and B). We can estimate that approximately 60% of this plasma tPA comes from hepatocytes (~40% hepatocyte-tPA silencing led to a 25% reduced plasma tPA protein; Figure 1A) and that approximately 70% of plasma PAI-1 is hepatocyte derived (Figure 1F). Importantly, plasma tPA was reduced in the hepatocyte–PAI-1 KO obese mice (Figure 3A), indicating that the PAI-1/*PLAT* pathway is an important contributor to the increase in hepatocyte-derived plasma tPA in obesity. Interestingly, a SNP, rs2227667, located in the human *SERPINE1* intronic region, is associated with an increase in circulating tPA levels (52), and data extracted from the GTExPortal (GTExPortal.org) suggests that this SNP may also be associated with higher *SERPINE1* in human livers. If these observations are confirmed in future cohorts, they may provide genetic evidence for the PAI-1/tPA pathway described herein.

In addition to affecting systemic fibrinolysis in obesity, regulation of fibrinolysis by hepatocytes may have other important implications in both disease and normal physiology. For example, obesity markedly increases the risk for chronic liver disease by promoting the development of NASH (53), and patients with liver disease are susceptible to both local thrombosis (54, 55), i.e., in the portal vein, and to systemic thrombosis (56–59). In terms of possible relevance to normal physiology, acute fluctuations in hepatic PAI-1 expression, e.g., in response to a meal or during infection (60–62), might require a compensatory increase in tPA to prevent impaired fibrinolysis. This response may then become compromised by disease-related processes, as exemplified by the elevation of DACH1 in hepatocytes in obesity, which decreases tPA expression and thereby limits the compensatory response. Interestingly, we showed previously that increased hepatocyte DACH1 in obesity also promotes excessive hepatic glucose production (32). Thus, hepatocyte DACH1 emerges as an important link between obesity-associated metabolic stress and impaired fibrinolysis. Accordingly, suppression of DACH1 using hepatocyte-targeted siRNA strategies, a modality that has been approved for use in humans (63), may provide an integrated approach to the problem of hepatocyte-mediated perturbations in metabolic disease.

The hepatocyte PAI-1/tPA pathway described here is unique when considering the pathophysiology of clotting disorders in obesity because hepatocytes can be considered to be at the intersection of sensing obesity-induced metabolic stress and regulating fibrinolysis. Thus, understanding this pathway may inform new strategies for improving basal fibrinolysis in obese subjects before an injury occurs. However, these strategies would have to take into account other perturbations associated with obesity, notably those related to coagulation and platelet function (64–69), which also contribute to the high risk of thrombotic disorders in the obese population (50, 70–72). Accordingly, the findings herein will likely have to be integrated with understanding of these other processes to fully define the pathophysiology of thrombotic disease in obesity and to conceive new therapies.

## Methods

### Mouse models.

WT mice used for silencing hepatocyte tPA (Figure 1A) and expressing hepatocyte Rev-Erbα (Figure 4C) were purchased from The Jackson Laboratory (JAX) as age-controlled lean and DIO mice (catalog 000664 and 380050, respectively). JAX DIO mice are available only as male mice. Both lean and obese mice were purchased at the age of 15 weeks and maintained with the same chow or DIO diet (Research Diets, catalog 12492) after transferring to the animal facility at Columbia University Irving Medical Center. After 1 to 2 weeks of adjustment to the new environment, AAV8 viruses were injected into the DIO mice when they were 17 weeks old. Experiments were started 3 weeks after the AAV8 injection. The mice were euthanized at the age of 22 to 23 weeks, so that the total time of DIO diet feeding was 4 months. The AAV8 constructs used were AAV8-H1-sh*Plat* (13), to silence hepatocyte *Plat*, and AAV8-TBG-Nr1d1 (73), to express hepatocyte Rev-Erbα. AAV8 vectors were delivered at 1 × 10^11^ genome copies/mouse. For AAV8-TBG-Nr1d1, the dose we used was 10% of the viral dose used in the original publication (73) to avoid excess expression. The AAV8-TBG-Nr1d1 vector was provided by Mitchell Lazar (University of Pennsylvania, Philadelphia, Pennsylvania, USA). Control mice were age-matched WT mice injected with AAV8-H1-LacZ and kept in the same animal facility for the same period.

The *Serpine1^fl/fl^* mice (Figure 1, F–I), *Dach1^fl/fl^* mice (Figure 2A), and *Creb1^fl/fl^* mice (Figure 6) were housed in the animal facility at Columbia University Irving Medical Center from birth. They were placed and maintained on a DIO diet (Research Diets, catalog 12492) at 6 weeks of age. The same DIO protocol with the same DIO diet, followed by the same AAV8 viral injection timing protocol, was used as above. Both male and female mice were used in these cohorts. Specifically, *Serpine1^fl/fl^* mice were generated and crossed onto the C57BL/6J background, as previously described (28). The mice were injected intravenously with AAV8-TBG-Cre at 4 months of age to delete PAI-1 in hepatocytes (13, 29–32, 74) (HC–PAI-1–KO mice). Control mice were *Serpine1^fl/fl^* mice injected with the AAV8-TBG-LacZ virus. *Dach1^fl/fl^* mice were generated as previously described (75) and crossed onto the C57BL/6J background. The mice were injected intravenously with AAV8-TBG-Cre at 4 months of age to deplete DACH1 in hepatocytes (13, 29–32, 74) (HC-DACH1–KO mice). Control mice were *Dach1^fl/fl^* mice injected with the AAV8-TBG-LacZ virus. The *Dach1^fl/fl^* mice were provided by Richard Pestell (Baruch S. Blumberg Institute, Doylestown, Pennsylvania, USA, and Nanyang Technological University, Singapore). AAV8 vectors were delivered at 1 × 10^11^ genome copies/mouse, and experiments were commenced 3 to 6 weeks later. *Creb1^fl/fl^* mice were generated as previously described (76) and crossed onto the C57BL/6J background. The mice were injected intravenously with AAV8-TBG-Cre at 4 months of age to delete CREB1 in hepatocytes (13, 29–32, 74) (HC-CREB1–KO mice). Control mice were *Creb1^fl/fl^* mice injected with the AAV8-TBG-LacZ virus. The *Creb1^fl/fl^* mice were provided by Eric J. Nestler (Icahn School of Medicine at Mount Sinai, New York, New York, USA).

For all experiments, mice were maintained on a 12-hour light/12-hour dark cycle with free access to normal chow or the DIO diet and water. Mice of the same age and similar weight were randomly assigned to experimental and control groups. Body weight was maintained throughout. Data for 5 weeks after AAV8 injection are shown in Supplemental Figure 6.

### Mouse thrombosis assays.

Carotid artery thrombosis was induced by either FeCl_3_-induced injury or by rose bengal/laser-induced photochemical injury, as previously described (77, 78). Briefly, mice were anesthetized with isoflurane and placed on a thermo-controlled blanket (37°C), followed by surgical exposure of the carotid artery. For the FeCl_3_ procedure, a filter paper soaked in 10% FeCl_3_ was applied to the artery for 3 minutes, followed by rinsing with normal saline. Blood flow was monitored with an ultrasound flow probe (Transonics) and recorded by LabChart software (ADInstruments). The time to total occlusion was defined as the interval between the application of FeCl_3_ and stable occlusion of the artery, defined as 0 blood flow for 3 minutes (77). For the rose bengal/laser procedure, 50 mg/kg of rose bengal in 0.15 mL of 0.9% saline was injected through the tail vein. A 540-nm wavelength laser light source (Melles Griot) held 6 cm away from the carotid artery was switched on to initiate photochemical injury, and then blood flow in the artery was monitored continuously (78). The time to total occlusion was defined as the interval between application of the laser and stable occlusion of the artery, with 0 blood flow for 10 minutes. After the procedure, the mice were immediately euthanized.

### Mouse tail-bleeding assay.

Mice were anesthetized with isoflurane and positioned horizontally on a platform that allowed the tail to descend approximately 2 cm from the top of the platform. A distal segment of the tail was transected with a no. 11 surgical scalpel to induce wounds of approximately 2-mm diameter. Bleeding time was monitored by gently dabbing the tail tip on Whatman paper at 10-second intervals until the cessation of bleeding (79). The time to stable cessation of bleeding was defined as the time interval between the tail incision and cessation of bleeding, with no evidence of rebleeding for 60 seconds. Bleeding exceeding 15 minutes was stopped by applying pressure.

### Plasma collection and analyses.

Blood obtained by cardiac puncture into 10% volume of sodium citrate (3.8%, w/v) was centrifuged for 15 minutes at 2300 *g*, and plasma was carefully collected from the supernatant fraction. Plasma samples were divided into aliquots, snap-frozen, and stored at −80°C until analyses. Plasma total antigen levels of tPA, PAI-1, and A2AP were measured by ELISA using kits according to the manufacturer’s instructions (catalog numbers are listed in Supplemental Methods). Plasma PAI-1–free tPA was measured by ELISA by first capturing the free tPA on a surface coated with PAI-1 antigen, enabling detection of only the functional, PAI-1–free form of tPA. tPA enzymatic activity in plasma or tissue lysates was assayed by chromatographically measuring the release of para-nitroaniline (pNA) chromophore from a plasmin-specific synthetic substrate (Abcam, ab108905). Results were recorded and analyzed by VersaMax Microplate Reader and SoftMax Pro software (Molecular Devices, Thermo Fisher Scientific).

### Euglobulin clot-lysis time.

A total of 50 μL of citrated mouse plasma was resuspended in 900 μL 0.017% acetic acid, placed on ice for 20 minutes, and then centrifuged for 20 minutes at 2000 *g* at 4°C. After careful removal of the supernatant fraction, each pellet (euglobulin fraction) was resuspended in 55 μL sodium borate/NaCl (pH 9.0) and transferred to a single well on a flat‑bottom 96-well microtiter assay plate; 50 μL of 25 mM CaCl_2_ was added to each well, and then the absorbance was recorded at 405 nm every 10 minutes, with 3-second shakes before each reading, at room temperature for 16 hours. Clot-lysis time was calculated as the time to achieve 50% of clot lysis (half-lysis time) (80).

### Human and mouse primary hepatocyte experiments.

Human primary hepatocytes were obtained from the Liver Tissue Cell Distribution System at the University of Pittsburgh (Pittsburgh, Pennsylvania, USA). Primary mouse hepatocytes were isolated from 10-week-old WT C57BL/6J mice, as described previously (32, 81). All cells were cultured in DMEM containing 10% FBS and then transfected with siRNAs described in the figure legends. The cells were then cultured in serum-free medium until they were harvested into RIPA buffer (Thermo Fisher Scientific, catalog 89900) with Halt Protease and Phosphatase Inhibitor Cocktail (Thermo Fisher Scientific, catalog 78444) for immunoblotting or into RNA lysis buffer (QIAGEN, catalog 79216) for mRNA quantification. Culture media were collected, snap-frozen in liquid nitrogen, and stored at –80°C until processing.

### Human liver specimens.

Human liver specimens were acquired from patients undergoing bariatric surgery or clinically indicated laparoscopic procedures at the New York Presbyterian Hospital, Columbia University Irving Medical Center (ref. 32, Figure 1E, Figure 2B, Figure 3D, and Supplemental Figure 3C). The liver specimens were from intraoperative needle biopsies and were frozen immediately in liquid nitrogen and stored at −80°C until subsequent analyses. The clinical characteristics of the individual donors appear in Supplemental Tables 1 and 2. We also received human liver specimens from the Liver Tissue Cell Distribution System at the University of Minnesota (Minneapolis, Minnesota, USA) (ref. 13 and Figure 2B). The specimens were collected postmortem on the date of liver transplantation and preserved as frozen samples.

### Statistics.

All results are presented as mean ± SEM. *P* values were calculated using 2-tailed Student’s *t* test for data that passed the normality test or the Mann-Whitney rank-sum *U* test for data that were not normally distributed. One-way ANOVA with post hoc Tukey’s test was used to evaluate differences among groups when 3 or more groups were analyzed.

### Study approval.

All mouse experiments were conducted with the approval of the IACUC of Columbia University Medical Center. The use of human cells and specimens in this study was approved by the IRB at the Columbia University Irving Medical Center. All participants provided written informed consent.

## Author contributions

ZZ and IT designed the research. ZZ, KN, SG, XW, and ST conducted the research. RML provided critical reagents related to PAI-1. ST, MB, BS, and AK organized patient recruitment and human liver sample collection. LO provided critical reagents and advice related to DACH1. ZZ, KN, SG, LO, JAL, and IT analyzed the data. ZZ, SG, JAL, and IT wrote the manuscript. All authors read and commented on the manuscript.

## Supplementary Material

Supplemental data

## Version 1 07/13/2020

Electronic publication

## Version 2 07/21/2020

Units corrected in Figure 1C

## Version 3 08/03/2020

Print issue publication
