# Supplementary material for: Interacting hepatic PAI-1/tPA gene regulatory pathways influence impaired fibrinolysis severity in obesity
Source: J Clin Invest. 2020 Jul 13;130(8):4348–59. doi: 10.1172/JCI135919 (PMC7410057; doi:10.1172/JCI135919)
Supplement: Supplemental data [file jci-130-135919-s300.pdf]

Supplemental Methods

Materials

Rose-Bengal	Fisher Scientific	Cat# R323-25
Sodium citrate (3.8%, w/v)	Fisher Scientific	Cat# 72284
Halt™ Protease and Phosphatase Inhibitor Cocktail	Thermo Fisher	Cat# 78444
RIPA buffer	Thermo Fisher	Cat# 89900
Halt™ Protease and Phosphatase Inhibitor Cocktail	Thermo Fisher	Cat# 78444
Protein G magnetic beads	Thermo Fisher	Cat# 88847
Supersignal West Pico ECL	Thermo Fisher	Cat# 34080
RNeasy Kit	Qiagen	Cat# 74106
QIAquick PCR Purification Kit	Qiagen	Cat# 28106
RNA lysis buffer	Qiagen	Cat# 79216
High-Capacity cDNA Reverse Transcription Kit	Thermo Fisher	Cat# 4374966
PowerUp SYBR Green Master Mix	Thermo Fisher	Cat# A25777
Rabbit anti-DACH1	Abcam	Cat# ab77234; RRID: AB_2277076
Rabbit anti-DACH1	Proteintech	Cat# 10914-1-AP; RRID: AB_2230330
Rabbit anti-Rev-Erbα	Cell Signaling	Cat# 13418; RRID:AB_2630359
Rabbit anti-pCREB1	Cell Signaling	Cat# 9198; RRID:AB_2561044
Rabbit anti-CREB1	Cell Signaling	Cat# 9197; RRID:AB_331277
Rabbit anti-PAI1	Abcam	Cat# ab66705; RRID:AB_1310540
Rabbit anti-GAPDH	Cell Signaling	Cat# 8884; RRID:AB_11129865
Rabbit anti-ACTB (β-actin)	Cell Signaling	Cat# 5125; RRID: AB_1903890
Rabbit normal IgG	Santa Cruz	Cat# sc-2025; RRID: AB_737182
Rabbit anti-HA	Santa Cruz	Cat# sc-7392; RRID: AB_627809
Rabbit anti-mouse tPA	Molecular Innovations	Cat# ASMTPA-GF-HT
Mouse anti-human tPA	Molecular Innovations	Cat# ASHTPA-GF-HT
Kinetic tPA activity kit	Abcam	Cat# ab108905
Mouse total tPA antigen ELISA	Molecular Innovations	Cat# MTPAKT-TOT

Mouse total PAI-1 antigen ELISA	Molecular Innovations	Cat# MPAIKT-TOT
Mouse total A2AP antigen ELISA	Molecular Innovations	Cat# MA2APKT-TOT
Human total tPA antigen ELISA	Molecular Innovations	Cat# HTPAKT-TOT
Human PAI1	Molecular Innovations	Cat# CPAI
Human PAI1, no LRP1 binding	Molecular Innovations	Cat# HPAI-R76E-I91L
H89 dihydrochloride, Kinase inhibitor	Calbiochem	Cat# 371962

Real-time quantitative-PCR. Total RNA was extracted from whole tissues or primary cells using the RNeasy Kit™ (Qiagen, 74106). cDNA was synthesized from 1 µg total RNA using the High-Capacity cDNA Reverse Transcription Kit™ (Thermo Fisher, #4374966). Real-time quantitative PCR (qPCR) was carried out using a 7500 Real-Time PCR system (Applied Biosystems) and PowerUp™ SYBR Green Master Mix (Thermo Fisher, #A25777). The sequences of the gene-specific primers used for the qPCR assays are listed in **Supplemental Table 3**. Fold changes in mRNA levels relative to a control condition were determined after normalization to the level of *Rplp0* mRNA.

Immunoblotting. Proteins from tissue or cell lysates were electrophoresed on 4-20% Tris-glycine gradient SDS gels and then transferred to nitrocellulose membranes. The membranes were blocked using either 5% non-fat milk or 5% BSA, followed by incubation with primary antibodies overnight. Blots were then washed thoroughly and probed with HRP-conjugated secondary antibodies for 1 hour. The protein bands were detected with Supersignal West Pico enhanced chemiluminescent (ECL) solution (Thermo Scientific, Cat. No. 34080). The densitometry of immunoblots was analyzed by Quantity One™ (Bio-Rad Laboratories).

Mouse liver nuclei preparation and ChIP assays. Mouse liver tissues were homogenized using a Dounce homogenizer (Wheaton, Cat#357544) with a loose pestle in 1:10 (w:v) of ice-cold NP-40 lysis buffer supplemented with protease inhibitor cocktail.

The liberation of nuclei was monitored by DAPI staining and fluorescence microscopy. To purify the intact nuclei, lysates were then layered over 1M (bottom) and 0.68M (top) of sucrose and spun at 4000 rpm for 30 min at 4°C. Following a washing step, nuclear pellets were cross-linked with 1% fresh formaldehyde in PBS for 10 min at room temperature. Cross-linking was terminated by adding 200mM Tris-HCl (pH 9.4) and 1mM DTT for 10 min and centrifuged at 2500 rpm for 15 min at 4°C. Nuclear pellets were suspended in SDS lysis buffer containing protease inhibitors, incubated for 10 min on ice, and sheared DNA in a cold water bath in a focused-ultrasonicator (Covaris, S2) to obtain DNA fragments with an average size of 500 bps. Fragmented chromatin was pre-cleaned by incubating with normal rabbit IgG for 1 hr at 4 °C, followed by 1 hr of incubation with 50 µL protein G magnetic beads (Pierce, Cat# 88847) at 4 °C with rotation. A rabbit anti-CREB1 antibody (Cat#9197) was used to pull down CREB1-binding complexes, and a control rabbit anti-HA antibody was used to pull down non-specific binding complexes. Immunoprecipitated chromatin fragments were reverse cross-linked, digested by proteinase K, and purified using QIAquick PCR Purification Kit (Qiagen, Cat# 28106). The presence of CREB1 in the *Plat* exonic region was quantified by qPCR and expressed relative to the input genomic DNA. The sequences of primers used for the ChIP-qPCR assays are described in **Supplemental Table 4**.

Table S1. Clinical characteristics of subjects whose specimens were assayed for plasma and liver PAI1 protein, liver *SERPINE1* and *PLAT* mRNA, and WAT SERPINE1 mRNA in Figure 1E, 3D, and Figure S3C.

#	BMI	Age	Gender	Diagnosis	Surgery	Medications
1	19.33	57	Female	Incisional hernia	Laparoscopic incisional hernia repair	Breoellipta inhalation, Creon 36000u, Nystatin 10000u/ml mouth/throat suspension, pantoprazole 40mg, ursodiol 300mg, ventolin HFA inhalation
2	22.66	66	Female	Cholelithiasis, moderate intrahepatic biliary dilatation	Laparoscopic cholecystectomy	Bupropion, Climara, Progesterone
3	22.76	71	Male	Colon cancer, impacted gallbladder	Lap right colectomy, lap cholecystectomy	Flagyl, polyethylene glycol, neomycin
4	25.99	78	Female	Gallbladder polyps & Cholelithiasis	Laparoscopic cholecystectomy	Prednisolone acetate 1% ophthalmic suspension, restasis 0.05% ophthalmic emulsion, synthroid citalopram
5	27.12	37	Female	Gallbladder polyps, mild hepatic steatosis	Laparoscopic cholecystectomy	Amoxicillin/ Clavulanate
6	27.83	31	Female	Cholelithiasis	Laparoscopic cholecystectomy	Albuterol sulfate NEBU, Colace CAPS, Cryselle, Iron Supplement, Prenatal Vitamins
7	32.48	30	Female	Cholelithiasis, suspected hepatic steatosis	cholecystectomy	
8	32.6	68	Female	Cholelithiasis	Lap cholecystectomy with intraoperative cholangiogram	Prozac 40mg (fluoxetine)
9	35	46	Female	morbid obesity, hepatic steatosis, hepatomegaly	Laparoscopic sleeve gastrectomy	HCT2, Losartan, Metoprolol
10	36.2	47	Male	Cholelithiasis	Laparoscopic cholecystectomy	Was on lipno and netronicb2ob for 14 days, 10 days before surgery
11	36.47	25	Female	Morbid obesity, pre-diabetes	Laparoscopic gastrectomy sleeve	Levothyroxine sodium 100mcg, metoprolol 25mg, niferex, vitD 50000u 1 capsule weekly, ferrous sulphate
12	36.7	67	Male	Morbid Obesity, symptomatic cholelithiasis, s/p lap Band, liver fatty infiltration, type 2 diabetes	Lap Sleeve Gastrectomy, removal of band, cholecystectomy	Metformin, Simvastatin, ASA 81mg, Hydroxychloroquine, Lipitor, Symbicort.
13	38.49	57	Female	Morbid obesity, type 2 diabetes	Gastric bypass, removal of gastric band	Aspirin 81mg, Baclofen 10mg, Topiramate, Toradol intramuscular (im), Trazodone,

						Klonopin, Valacyclovir, Albuterol, Keppra, Metformin, Xyzal, Dihydroergotamine, Thorazine
14	40.62	44	Female	Morbid obesity	Laparoscopic gastric bypass	Aripiprazole, Calcium citrate, Lorazepam, wellbutrin XL150mg, zoloft, zyrtec
15	41.94	43	Female	Morbid obesity, hepatic steatosis, hepatomegaly	Laparoscopic sleeve gastrectomy	atorvastatin, metformin, vitD 50000u
16	42	50	Female	morbid obesity, hepatic steatosis	Laparoscopic sleeve gastrectomy	Enalapril, Fluoxetine, Bupropion, Ibuprofen
17	43.91	55	Female	Morbid obesity, Steatohepatitis, with moderate necroinflammatiory activity, no fibrosis, pre-diabetes	Laparoscopic gastrectomy Sleeve & repair of hiatal hernia	Allegra, Meclizine, VitD
18	44.45	24	Female	Morbid obesity	Laparoscopic sleeve gastrectomy, Hiatal hernia repair	vitD 50000u
19	44.48	26	Male	Morbid obesity	Laparoscopic sleeve gastrectomy	Lamotrigine, Intuniv
20	45.48	54	Male	Morbid obesity, type 2 diabetes	Laparoscopic sleeve gastrectomy	Edarbyclor, Furosemide 40mg, Lantus 100u/mL, Metformin 1000mg, Metoprolol succinate ER 25mg s/c, Novolog 100u/ml s/c, Pantoprazole, Strovite, Valacyclovir, VitD
21	45.66	58	Female	Obesity, type 2 diabetes	Lap Gastric Bypass	Amphetamine-dextroampheter, bupropion Hcl ER (XL), Furosemide, Ondansetron, Pravachol 40mg, Synthroid, VitD, Valsartan, Metformin, Aspirin
22	45.73	25	Female	Morbid obesity, moderate hiatal hernia	Laparoscopic sleeve gastrectomy, Hiatal hernia repair	MultiVit, Ca2++
23	48.3	52	Female	Morbid obesity, hepatic steatosis, type 2 diabetes	Laparoscopic Gastric Bypass	Omeprazole, Lisinopril, Singulair, Proventil HFA, Atorvastatin, Chlorthalidone, Advair Diskus, Topiramate
24	50.32	28	Male	Morbid obesity, hepatic steatosis, type 2 diabetes	Lap Gastrectomy Sleeve	Aspirin 81mg, calcium, Levetiracetam, Lisinopril, Metformin, Vitamin D.
25	59.02	34	Male	Morbid obesity, mild steatosis, predominantly large droplet fat, no increase in fibrosis	Laparoscopic sleeve gastrectomy	Omeprazole, VitD

Table S2. Clinical characteristics of subjects whose livers were assayed for liver DACH1 protein and plasma tPA activity in Figure 2B, left and middle panels

#	BMI	Liver DACH1/β-actin	Diagnosis of diabetes	Medication for diabetes
1	18.7	1.0	No	No
2	19.7	1.6	No	No
3	20.7	3.6	No	No
4	21.9	8.8	No	No
5	24.3	5.6	Yes	No
6	26.8	3.2	No	No
7	29.4	3.2	No	No
8	29.5	9.3	No	No
9	30.3	9.4	No	No
10	37	5.0	Yes	Sitagliptin
11	51.6	8.9	No	No
12	52.5	13.8	Yes	Insulin
13	53	15.1	Prediabetic	Metformin
14	62	19.4	Yes	Metformin

Table S3. qPCR primers for mRNA assays.

#	Genomic region	Direction	5'- Sequence -3'	Organism
1	*Rplp0*	forward	GCTCCAAGCAGATGCAGCA	mouse
2	*Rplp0*	reverse	CCGGATGTGAGGCAGCAG	mouse
3	*Plat*	forward	AGCACTCTCGGGACACAGAA	mouse
4	*Plat*	reverse	GTCTGCGTTGGCTCATCTCT	mouse
5	*Serpine1*	forward	CAATGGAAGGGCAACATGACC	mouse
6	*Serpine1*	reverse	AGCTGCTCTTGGTCGGAAA	mouse
7	*Nr1d1*	forward	ctactggctccctcacccagga	mouse
8	*Nr1d1*	reverse	gacactcggctgctgtcttcca	mouse
9	*Creb1*	forward	ATCTGGAGCAGACAACCAGC	mouse
10	*Creb1*	reverse	GGCATGGATACCTGGGCTAA	mouse
11	*Lrp1*	forward	atcaactggcggtgtgacaa	mouse
12	*Lrp1*	reverse	ggtcttgtagcctggttggt	mouse
13	*RPLP0*	forward	CGTCCTCGTGGAAGTGACAT	human
14	*RPLP0*	reserve	CTTGGAGCCCACATTGTCTG	human
15	*PLAT*	forward	gtttcgcccagccaggaaat	human
16	*PLAT*	reverse	tattccacccggttgcttct	human
17	*SERPINE1*	forward	GCGCTGCAGAAAGTGAAGAT	human
18	*SERPINE1*	reverse	AAGGACTGTTCCTGTGGGGT	human
19	*NR1D1*	forward	TCATGCTTGCGAAGGCTGTA	human
20	*NR1D1*	reverse	CGACCAAACCGAACAGCATC	human
21	*CREB1*	forward	GTGACGGAGGAGCTTGTACC	human
22	*CREB1*	reverse	CTGGCATAGATACCTGGGCT	human
23	*LRP1*	forward	GAGTCCAATGCCACTTGTTCAG	human
24	*LRP1*	reverse	CAGTCATTGTCATTGTCGCATCT	human

Table S4. ChIP-qPCR primers

#	Genomic region	Direction	5'- Sequence -3'	Organism
1	CREB1 consensus sequence in *Plat* promoter	forward	GCACCAAAAGTCATGTGCGT	mouse
2	CREB1 consensus sequence in *Plat* promoter	reverse	GCAACTACCCCTCAGGACAC	mouse
3	Rplp0 promoter	forward	ACTAATCGGGGGTGCAAACT	mouse
4	Rplp0 promoter	reverse	CTGTGATGCACAGGCAGTAT	mouse

Supplemental Figure Legends

Supplemental Figure 1. Diet-induced obese mice have increased plasma tPA, reduced plasma tPA activity, delayed clot lysis time and shortened time to arterial thrombotic occlusion. (**A-C**) Chow-fed mice (Lean) or diet-induced obese mice were assayed for the following parameters: (**A**) Plasma tPA protein concentration, plasma tPA activity, plasma fibrinolytic activity measured by the euglobulin clot lysis assay, and time to occlusive carotid arterial thrombosis induced by rose bengal-laser photochemical injury; (**B**) Plasma PAI-1 protein concentration; and (**C**) liver *Plat* mRNA levels and liver tPA activity. Horizontal lines in dot-density plots indicate mean values. Asterisks represent statistical significance at $^{**}P < 0.01$ using two-tailed Student's *t*-test (n = 8-10 mice per group). (**D**) *Plat* mRNA levels in the liver and endothelial layer of descending aortic artery from lean and obese WT mice. Asterisks represent statistical significance of $^{*}P < 0.05$, and n.s. indicates non-significance ($P > 0.05$), using two-tailed Student's *t*-test (n = 3 mice per group). (**E**) PAI-1 concentration in the plasma of the three groups of mice in Figure 1A-C. Asterisks represent statistical significance of $^{*}P < 0.05$ or $^{**}P < 0.01$, and n.s. indicates non-significance ($P > 0.05$), using one-way analysis of variance followed by Tukey's test (n = 9-10 mice per group). (**F**) A2AP concentration in the plasma of hepatocyte tPA-silenced obese mice (shPlat) and control AAV8-H1-Scr-treated obese mice (Ctrl); n.s. indicates non-significance ($P > 0.05$) using two-tailed Student's *t*-test (n = 9 mice per group). (**G**) *Serpine1* mRNA levels in the livers from lean and obese WT mice. The asterisk represents statistical significance of $^{**}P < 0.01$, using two-tailed Student's *t*-test (n = 10 mice per group). (**H**) PAI-1 protein levels in lysates of liver and visceral white adipose tissue (WAT) from lean and obese WT mice were measured by immunoblot, with GAPDH serving as the loading control (n = 3 mice per group). (**I**) A2AP concentration in the plasma of the two groups of mice in Figure 1F-I; n.s. indicates non-significance ($P > 0.05$) using two-tailed Student's *t*-test (n = 11 mice per group).

Supplemental Figure 2. Increased DACH1 protein in the livers of obese mice, and documentation of DACH1 depletion in the livers of *Dach1fl/fl* mice treated with

AAV8-TBG-Cre. (**A**) Immunoblot of DACH1 in the livers of lean and obese WT mice (n = 9-10 mice per group). (**B**) Immunoblot of DACH1 in the livers (n = 4 mice per group), and (**C**) plasma PAI-1 protein concentration of the 3 groups of obese *Dach1fl/fl* mice from Figure 2A, which were treated with combinations of AAV8-TBG-Cre versus AAV8-TBG-LacZ control virus and AAV8-H1-shPlat versus AAV8 control virus (Ctrl) (n = 5-7 mice per group). (**D**) Liver specimens from 19 wild-type mice with a wide range of body weight (lean and obese) were assayed for DACH1:β-actin densitometric ratio on immunoblot, and their plasma and liver lysate were assayed for tPA activity. The graphs show plots of the indicated correlations, which were analyzed by linear regression, with r^2 and *P* values indicated in the graph.

Supplemental Figure 3. The cell autonomous PAI-1 → *PLAT* pathway is conserved in primary mouse hepatocytes, and PAI-1 immunoblot in the livers of the human subjects analyzed in Figure 3D. (**A**) Primary mouse hepatocytes were treated for 16 hours with 100 μM palmitate in BSA solution, or BSA solution control. The cells were assayed for *Plat* and *Serpine1* mRNA, and the media were assayed for tPA and PAI-1 protein concentration. (**B**) Mouse primary hepatocytes were transfected with si-Serpine1 or scrambled control (Scr). After 24 hours, the cells were treated with 100 μM palmitate for an additional 16 hours and then assayed for *Plat* and *Serpine1* mRNA. In A and B, results are shown as mean ± SEM. Asterisks represent statistical significance at $^*P < 0.05$ or $^{**}P < 0.01$ using two-tailed Student's *t*-test (n = 3 sets of cells per group). (**C**) Immunoblot of PAI-1 in the livers of the 25 human subjects analyzed in Figure 3D and described in Table S1.

Supplemental Figure 4. Further characterization of the mice treated with AAV8-TBG-Nr1d1 or control AAV8 in Figure 4C. Lean and obese mice were injected intravenously with AAV8-TBG-Nr1d1 or AAV8-TBG-LacZ control (LacZ), as indicated. After 4 weeks, the following parameters were measured: (**A**) Liver *Nr1d1* mRNA by qPCR (n = 10 mice per group) and Rev-Erbα protein by immunoblot (n = 4 mice per group); and (**B**) Plasma PAI-1 protein, plasma tPA activity, and plasma fibrinolytic activity assayed by the euglobulin clot lysis assay. Horizontal lines in the dot-density

plots indicate mean values. Asterisks represent statistical significance of *$P < 0.05$ or **$P < 0.01$, and n.s. indicates non-significance ($P > 0.05$), using a one-way analysis of variance followed by Tukey's test (n = 10 mice per group). (**C**) Human primary hepatocytes were transfected with scrambled RNA control (Scr), siNR1D1, siSERPINE1, or both siRNAs. After 24 hours, the cells were treated with 100 μM palmitate for 16 hours and then assayed for *SERPINE1, PLAT, and NR1D1* mRNA. Results are shown as mean ± SEM. The asterisk represents statistical significance at *$P < 0.05$, and n.s. indicates non-significance ($P > 0.05$), using one-way analysis of variance followed by Tukey's test (n = 3 sets of cells per group).

Supplemental Figure 5. Documentation of *CREB1* silencing in the human hepatocytes analyzed in Figure 5D-E; conservation of the PAI-1-CREB-Plat pathway in primary mouse hepatocytes; and ChIP control for Figure 5F. (**A**) Immunoblots of DACH1 in the livers of obese *Serpine1fl/fl* mice from Figure 1E-H. For the left blot, obese mice were treated with AAV8-TBG-LacZ or AAV8-TBG-Cre, (n = 5 mice per group, left panel). For the right blot, the livers from lean and obese mice were injected intravenously with AAV8-TBG-Nr1d1 or AAV8-TBG-LacZ control (LacZ) (n = 4 mice per group, right panel). (**B**) Human primary hepatocytes were transfected with siCREB1 or scrambled control. After 24 hours, the cells were treated for 8 hours with 1 μg active recombinant PAI-1 (rPAI-1) per mL culture medium or vehicle control (Veh). The cells were then assayed for *CREB1* mRNA. (**C**) Mouse primary hepatocytes were transfected with siCreb1 or scrambled control (Scr). After 24 hours, the cells were treated for 8 hours with 1 μg recombinant PAI-1 (rPAI-1) per mL culture medium or vehicle control (Veh). The cells were then assayed for *Plat and Creb1* mRNA. For A and B, the results are shown as mean ± SEM. Asterisks represent statistical significance of *$P < 0.05$, and n.s. indicates non-significance ($P > 0.05$), using a one-way analysis of variance followed by Tukey's test (n = 3 sets of cells per group). (**D**) Nuclear extracts from the livers of lean or obese mice were subjected to ChIP assay using anti-CREB1 or control IgG, as in Figure 5F. The Rplp0 promoter region was amplified by qPCR and normalized to the values obtained from input DNA. Results are shown as mean ± SEM; n.s. indicates non-significance ($P > 0.05$) using one-way analysis of variance followed

by Tukey's test (n = 3 mice per group). (**E**) Plasma samples from control and Cre-treated *Creb1fl/fl* mice from Figure 6 were assayed forPAI-1 concentration. Horizontal lines in dot-density plots indicate mean values; n.s. indicates non-significance ($P > 0.05$) using two-tailed Student's *t*-test (n = 8-9 mice per group). (**F**) Plasma samples from the 3 groups of mice in Figure 7C were assayed for PAI-1 concentration. Asterisks represent statistical significance at $^{**}P < 0.01$ and n.s. indicates non-significance ($P > 0.05$) using one-way analysis of variance followed by Tukey's test (n = 5 mice per group).

Supplemental Figure 6. Documentation of mouse body weight gain for all in vivo cohorts in the manuscript. (**A**) The change of body weight of individual mice from the time of AAV8 vector injection (open circles) to the time of euthanasia (solid black dots). (**B**) The body weight gain of each mouse was calculated as the difference in the body weight from the time of AAV8 vector injection to the time of euthanasia.

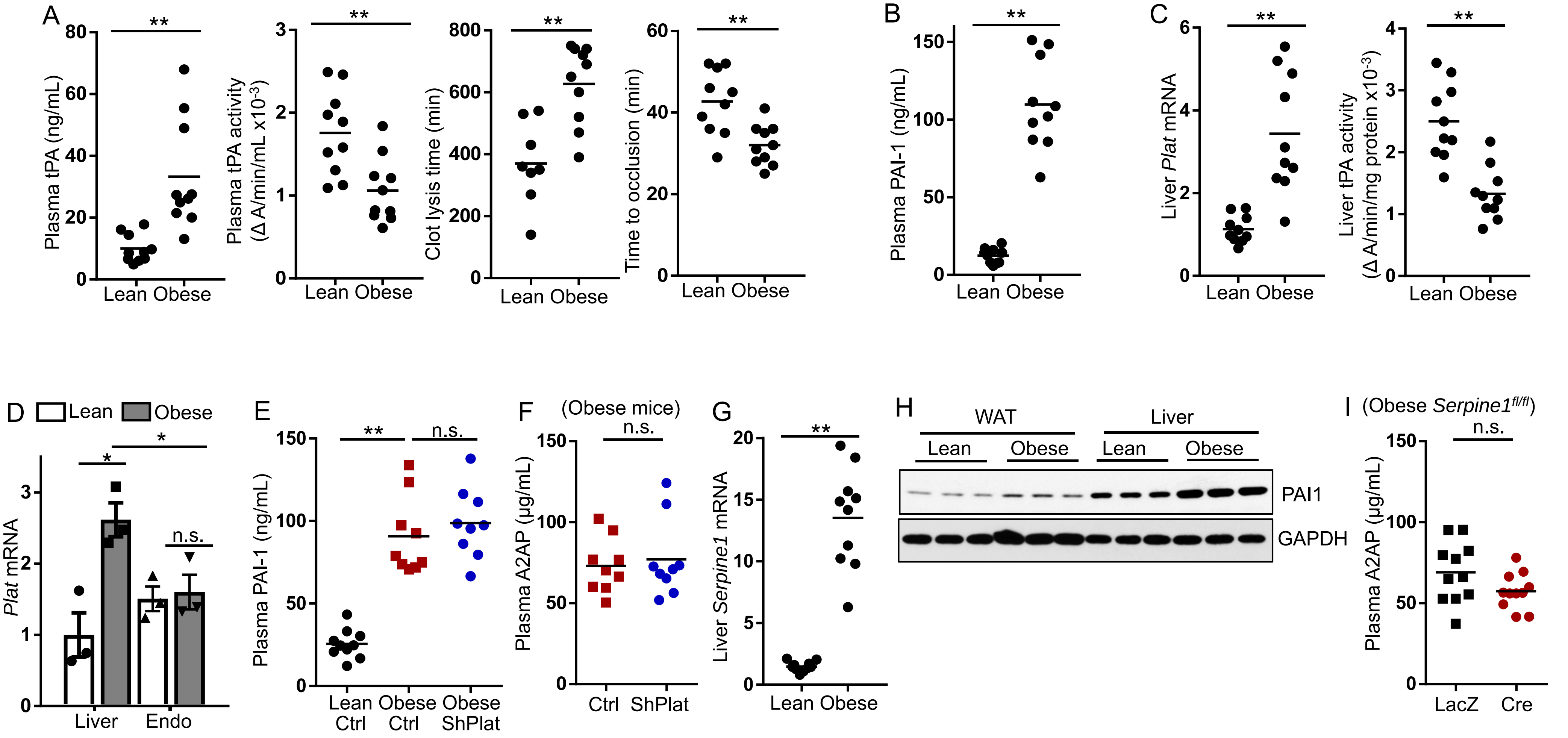

Supplemental Figure 1

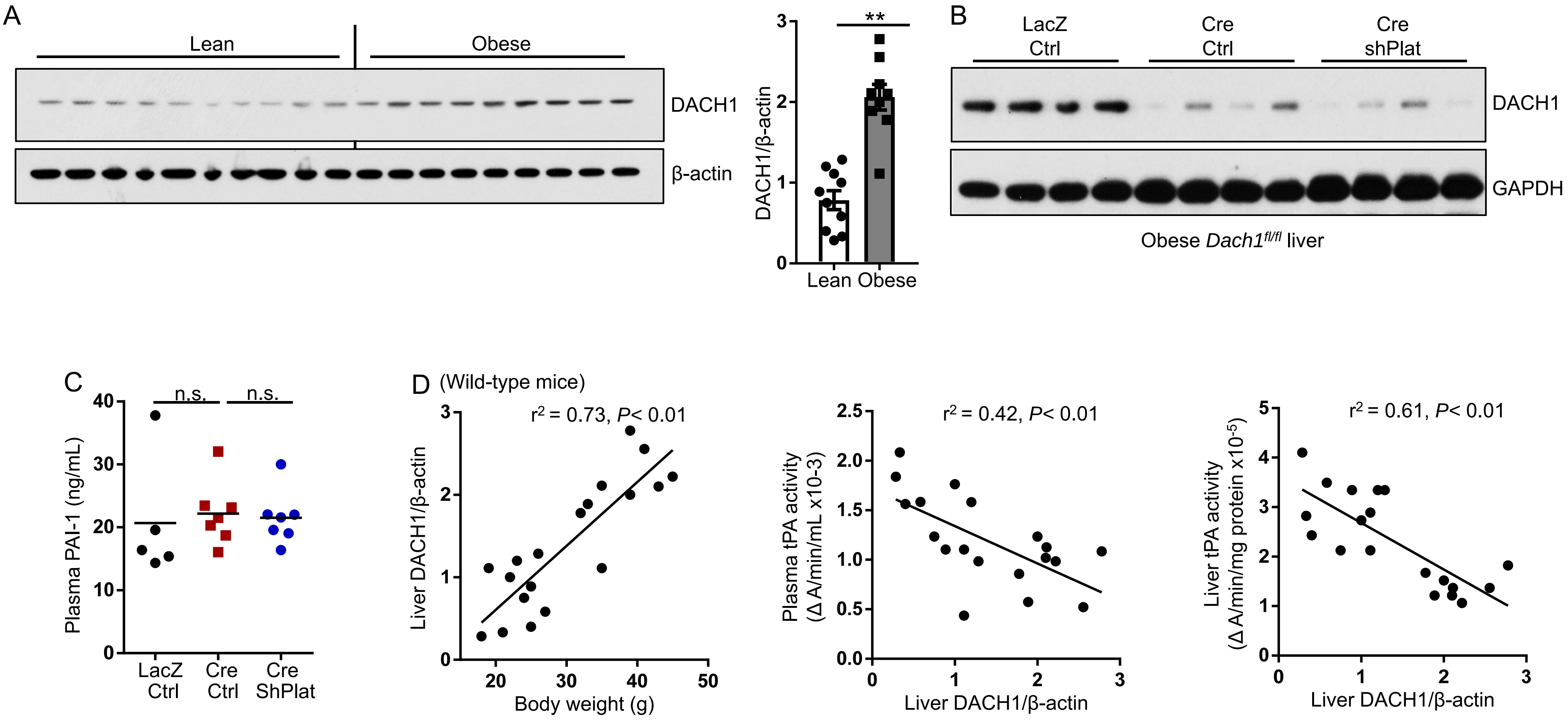

Supplemental Figure 2

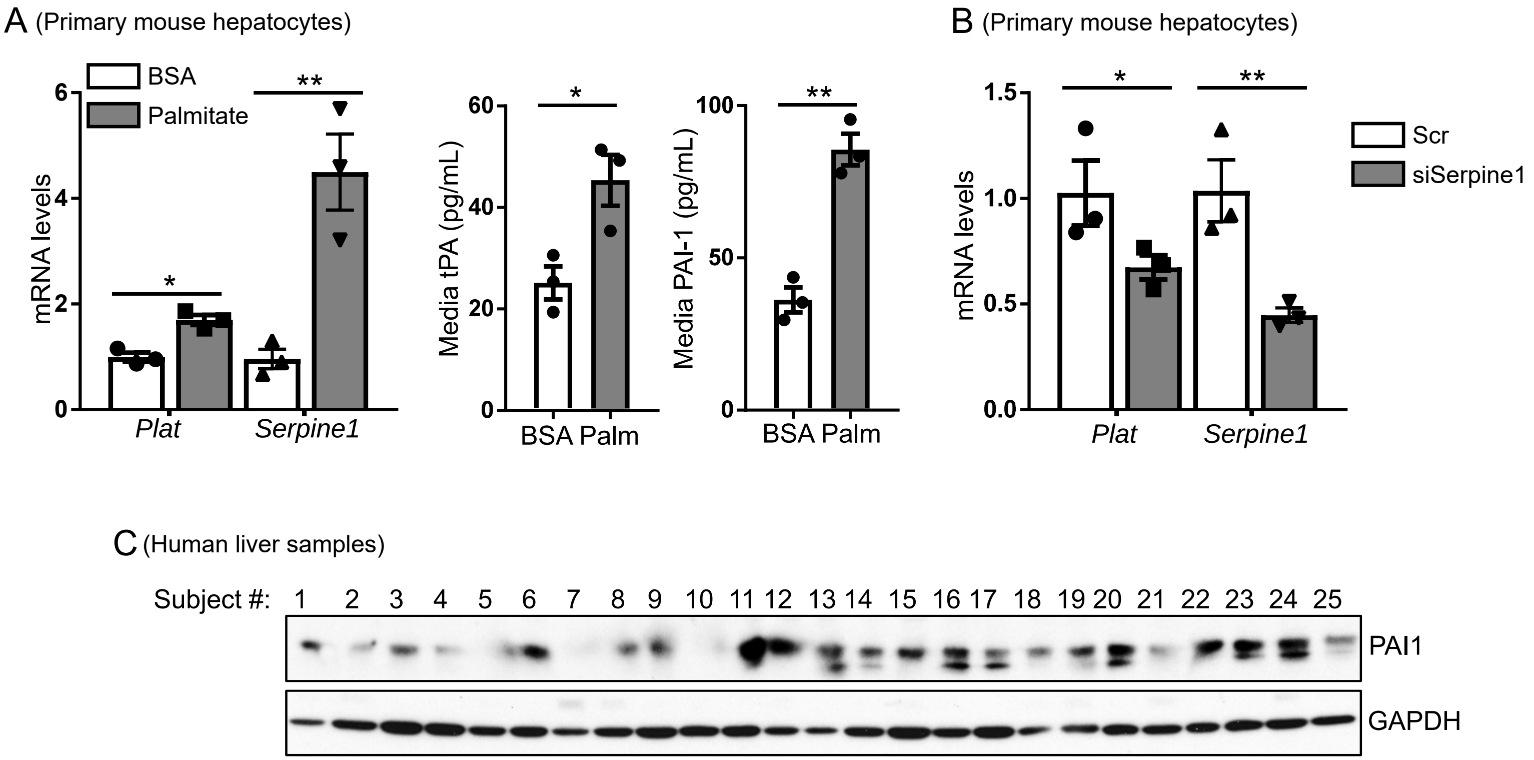

Supplemental Figure 3

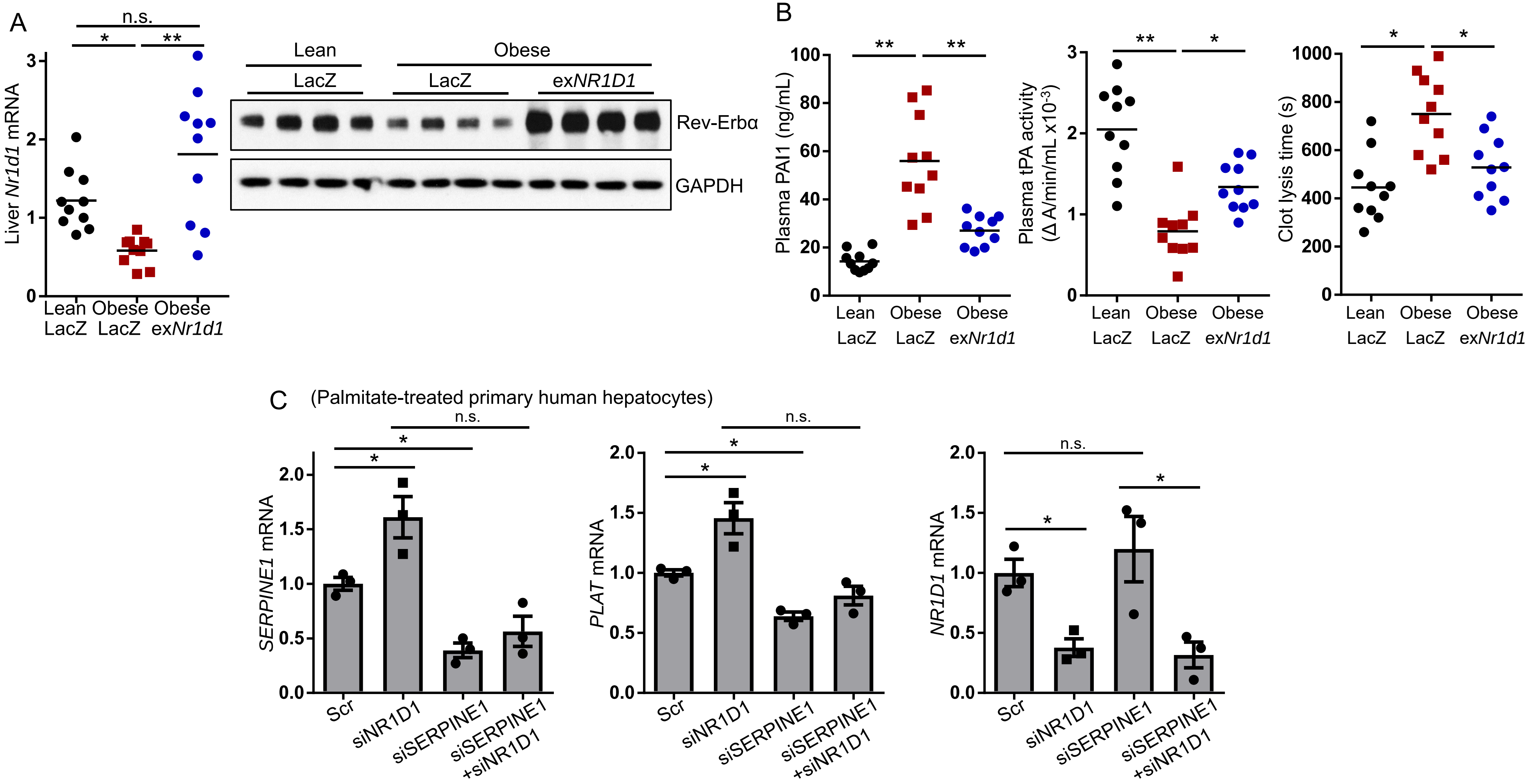

Supplemental Figure 4

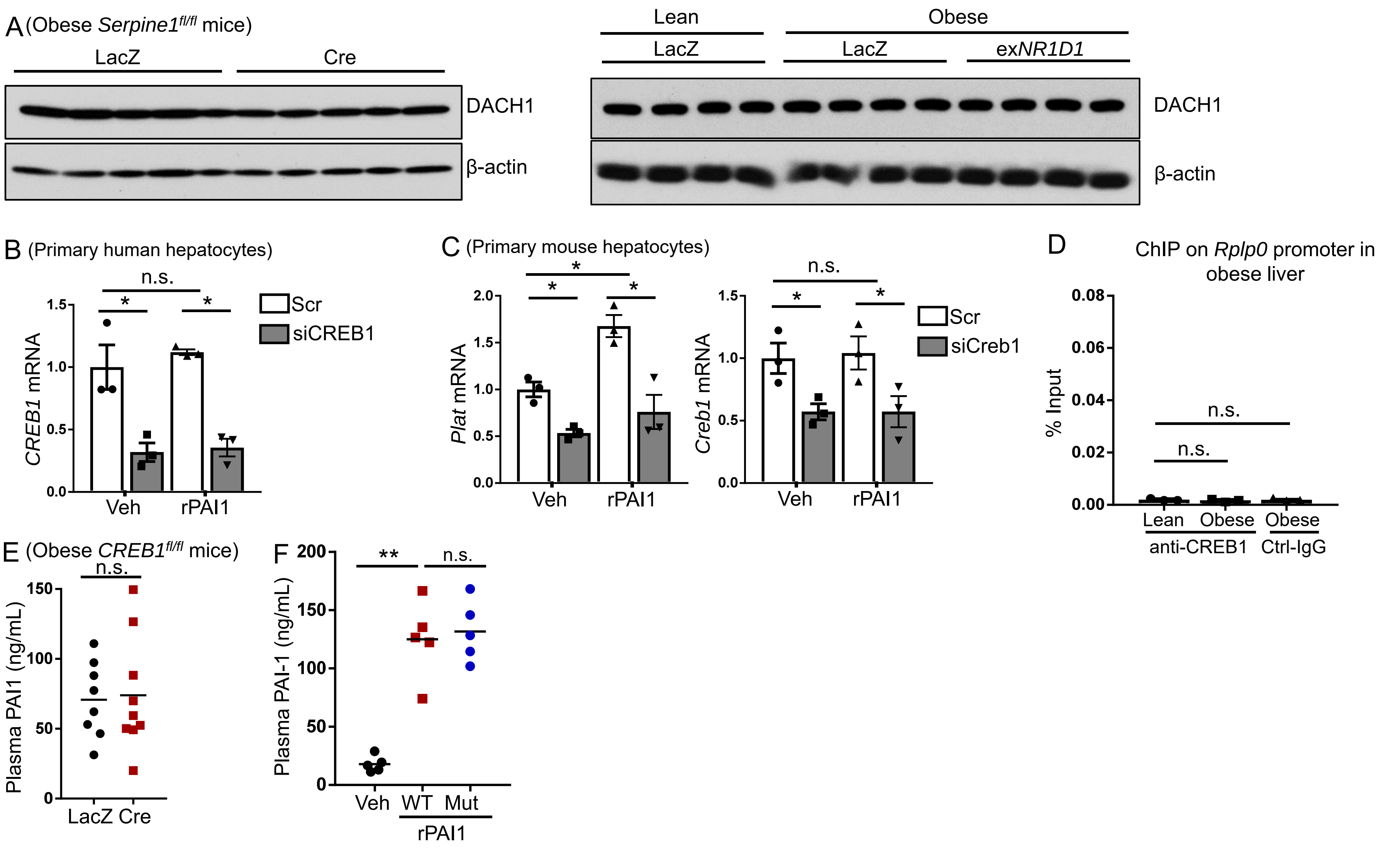

Supplemental Figure 5

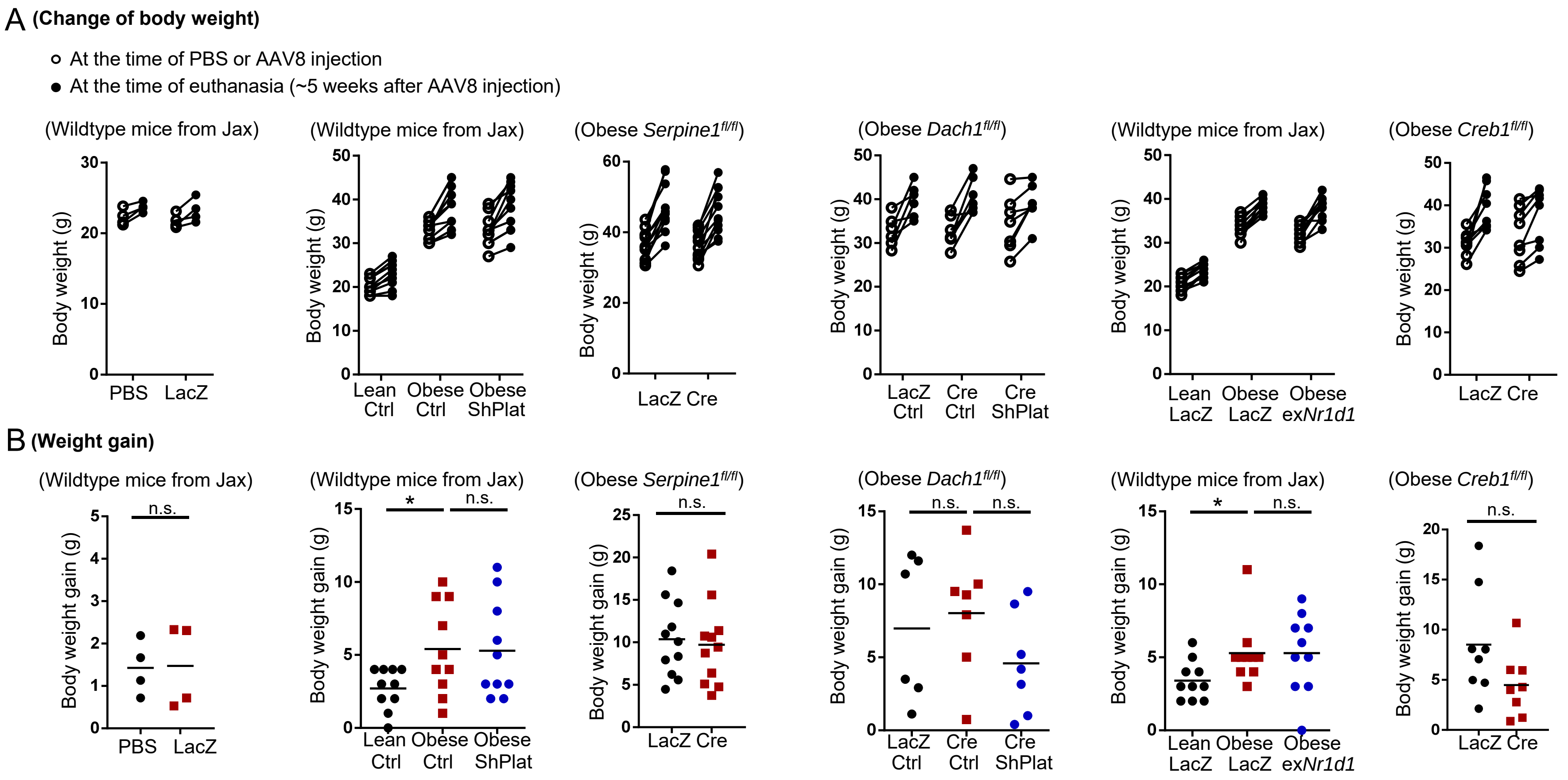

Supplemental Figure 6
